# Cyanobacterial Biofactories Beyond Model Strains: Exploratory Screening of Immunomodulatory Activity in *Phormidium ambiguum* Extracts

**DOI:** 10.3390/plants15010033

**Published:** 2025-12-22

**Authors:** Sandugash K. Sandybayeva, Shakhira A. Ismailova, Anna O. Yershova, Ardak B. Kakimova, Bolatkhan K. Zayadan

**Affiliations:** 1 Department of Biotechnology, Faculty of Biology and Biotechnology, Al-Farabi Kazakh National University, 71 Al-Farabi Ave., Almaty 050040, Kazakhstan; 2 Department of Chemical and Biochemical Engineering, Geology and Oil-Gas Business Institute Named after K. Turyssov, Satbayev University, 22 Satbaev Str., Almaty 050013, Kazakhstan

**Keywords:** cyanobacteria, antioxidant activity, immunomodulation, antibacterial activity, bioactive metabolites, biotechnological potential, high value-products

## Abstract

Photosynthetic prokaryotes known as cyanobacteria produce an extensive range of primary and secondary metabolites that serve multiple biotechnological and biomedical purposes. The non-model filamentous *Phormidium* species capture researchers’ attention through their biotechnological potentials from diverse metabolites and their ability to thrive in tough environments while producing bioactive compounds. In this study, a thermotolerant strain of *Phormidium ambiguum* was isolated from the Chundzha thermal springs in southeastern Kazakhstan and characterized morphologically, physiologically, and biochemically. This cyanobacterium demonstrated fast growth in its culture media along with significant accumulation of proteins (44.8% DM), carbohydrates (45% DM), and photosynthetic pigments like chlorophyll a and valuable carotenoids, including b-carotene, myxoxantophyll and zeaxanthin. The LC-ESI-MS/MS analysis of cyanobacterial non-polar extract identified 150 potential metabolites which include fatty acid derivatives, terpenoids and carotenoid-related compounds known for their antioxidant and antimicrobial properties, as well as immune system stimulation. Biological assays confirmed a weak antioxidant capacity in the DPPH test, while in immunological assays, the extract of *P. ambiguum* stimulated T lymphocyte proliferation and activation, as well as NK cell proliferation in vitro. It also exhibited moderate antibacterial activity towards tested Gram-negative and Gram-positive bacterial strains. While additional studies are required to address environmental robustness, biosynthetic regulation, and practical scalability, the present findings indicate that *P. ambiguum* represents a valuable non-model cyanobacterium for exploratory bioprospecting. Its metabolite profile and observed bioactivities support further investigation of this thermotolerant strain as a potential source of immunomodulatory, antioxidant, and antimicrobial compounds under controlled conditions.

## 1. Introduction

The transition toward a sustainable bioeconomy increasingly depends on the utilization of renewable biological resources to produce food, energy, and materials in an environmentally responsible manner [1]. However, the global demand for food and bio-based commodities continues to rise amid the dual constraints of limited arable land and accelerating climatic stress, rendering further agricultural expansion both economically inefficient and ecologically undesirable. Large-scale cultivation of terrestrial plants for functional ingredients is further hindered by long growth cycles, low productivity, and the resource-intensive extraction of valuable compounds [2]. Moreover, deforestation, bioprospecting restrictions, and concerns over biopiracy challenge the ethical and sustainable use of plant resources for industrial purposes [3]. These limitations have prompted an intensified search for alternative biological platforms capable of producing high-value products more efficiently and with lower environmental impact. In this context, photosynthetic microorganisms—particularly cyanobacteria—have emerged as promising candidates for next-generation biofactories due to their remarkable adaptability and rich metabolic repertoire. They combine key physiological features of higher plants (efficient photosynthesis, oxygen evolution, autotrophic nutrition) with microbial advantages such as rapid growth, high productivity, and the capacity to accumulate or secrete bioactive compounds [4,5]. These organisms are found in nearly all environments on Earth—from freshwater and marine ecosystems to hot springs, deserts, and polar ice—demonstrating extraordinary ecological plasticity [6]. This adaptability allows cyanobacteria to produce structurally and functionally diverse bioactive molecules, including pigments, lipids, polysaccharides, vitamins, and secondary metabolites with antioxidant, antimicrobial, or immunomodulatory properties [7,8].

The economic potential of cyanobacteria spans multiple industrial sectors, including the production of biofuels, bioplastics, pharmaceuticals, cosmetics, nutraceuticals, and sustainable food and feed ingredients [9]. To date, more than 1600 structurally unique compounds have been identified from cyanobacterial sources, encompassing lipopeptides, alkaloids, macrolides, indoles, amides, and polysaccharides, many of which display potent pharmacological properties [10,11,12]. Some cyanobacterial compounds or their semisynthetic analogues, such as cryptophycin derivatives and dolastatin-related peptides (LY 355073, oxazoline, TZT-1027, cematodin, and synthadotin), have already advanced into clinical evaluation as potential anticancer agents [13,14]. In addition to secondary metabolites, cyanobacteria produce a diverse array of pigments such as phycobiliproteins, chlorophylls, and carotenoids that not only function as essential light-harvesting molecules but also exhibit antioxidant, anti-inflammatory, anti-cancer, neuroprotective, anti-obesity and immunomodulatory properties [15,16,17]. Recent studies have demonstrated that phycocyanin and carotenoid-rich extracts from cyanobacteria can modulate cytokine production, enhance macrophage function, and attenuate inflammatory responses, highlighting their potential as natural immunotherapeutic agents in nutraceutical and biomedical applications [18,19].

Despite intensive research on model strains such as *Synechocystis* sp. PCC 6803 and *Synechococcus* sp. PCC 7002, the majority of the approximately 150 recognized cyanobacterial genera—comprising more than 2600 species—remain insufficiently characterized [20]. Although these models are invaluable for fundamental studies, their large-scale cultivation often proves inefficient due to sensitivity to fluctuating environmental conditions and metabolic limitations. Consequently, attention is shifting toward non-model filamentous cyanobacteria, including *Phormidium*, *Oscillatoria*, and *Leptolyngbya*, which are naturally adapted to extreme habitats and capable of producing thermostable and halotolerant biomolecules. These resilient strains hold great promise for industrial-scale photobioprocesses, where robustness and high productivity under variable conditions are key [21,22,23]. Among them, the genus *Phormidium* has recently attracted growing interest as a source of novel bioactive compounds and thermostable pigments suitable for food, pharmaceutical, and cosmetic applications [24,25]. Exploring such non-model cyanobacteria not only broadens our understanding of their physiological and biochemical diversity but also contributes to the development of new sustainable platforms for bioproduct synthesis and circular bioeconomy.

*Phormidium*, a fast-growing filamentous cyanobacterium, has recently attracted attention as a versatile biological platform for sustainable biotechnological applications. Its natural abundance in aquatic and extreme environments, coupled with the ability to synthesize a wide spectrum of high-value metabolites positions it as an economically promising candidate for integrated biorefineries [26]. In non-sterile outdoor photobioreactor conditions, *Phormidium* species demonstrate consistent growth and are considered appealing candidates for industrial bioproduction [27]. Filamentous growth is another feature that might be useful for various biotechnological applications that offers easier separation from growth medium, potential for high cell densities in biofilm formation, and continued motility for specific applications [28]. Nevertheless, the transition from laboratory-scale cultivation to large-scale production introduces considerable technical and economic challenges. Industrial cyanobacterial cultivation typically relies on open ponds, closed ponds, photobioreactors (PBRs), or biofilm-based platforms, each presenting its own set of limitations [29]. In addition to system design, resource availability and cost significantly influence process feasibility. Scaling cyanobacterial biomass production requires substantial water and nutrient inputs, and traditional culture media are not economically viable for high-volume operations. While the use of commercial sea salts, natural seawater, or agricultural fertilizers can reduce medium costs, sometimes by more than 90%—these substitutes may lack essential micronutrients or require additional supplementation to maintain productivity [30,31]. Wastewater-based cultivation offers an alternative route to mitigate nutrient expenses, though it also introduces risks of contamination and unintended metabolite formation, particularly for strains intended for food or biomedical applications [32]. Maintaining culture stability and preventing growth collapse remain central challenges, often necessitating advanced monitoring systems that significantly increase operational costs [33]. Despite these limitations, cyanobacteria, including *Phormidium* spp., retain several intrinsic advantages that sustain their potential within targeted biotechnological frameworks. Their ability to grow phototrophically using sunlight, CO_2_, water, and minimal nutrients eliminates the need for expensive organic carbon sources and complex media. Sunlight, the most abundant renewable energy resource, enables the conversion of solar energy directly into biomass, providing a low-input, environmentally friendly basis for cultivation [34]. Cyanobacteria can convert up to 39% of available solar energy into biomass—far exceeding the ≤0.25–3% observed for conventional crops such as maize and sugarcane [15]. Their high growth rates, capacity for continuous cultivation, and potential compatibility with non-arable land or wastewater streams further expand the range of feasible production environments.

The pigment profile of *Phormidium* species, comprising chlorophyll a, zeaxanthin, echinenone, β-carotene, and phycocyanin, underpins their commercial value as natural colorants and functional ingredients with demonstrated antioxidant and antimicrobial properties [35]. Several compounds of *Phormidium* origin have been shown to possess potent antiparasitic, cytotoxic, and anticancer activities. For instance, two novel natural products, hierridin A and hierridin B, were isolated from the lipophilic extract of the marine cyanobacterium *Phormidium ectocarpi* and demonstrated pronounced antiplasmodial activity against *Plasmodium falciparum*, highlighting the potential of *Phormidium* metabolites as leads for antimalarial drug development [36]. Likewise, Dzhambazov et al. reported the specific antitumour activity of two *Phormidium molle* strains, whose extracts exhibited selective cytotoxic effects toward human cancer cell lines (HeLa and A2058) while maintaining relatively low toxicity to normal fibroblast cells [37]. Treatment with *Phormidium* extracts induced cytoskeletal and microtubule disorganization, resulting in a dose-dependent alteration in cell morphology and viability, thereby suggesting a mechanistic basis for their anticancer potential. Moreover, portoamides A and B, cyclic peptides isolated from *Phormidium* sp. LEGE 05292, were shown to exert cytotoxic activity against both normal epithelial (RPE-1) and colon carcinoma (HCT116) cells, further confirming the diverse chemical arsenal encoded within this genus [38]. Collectively, these findings underscore the wide-ranging bioactivities of *Phormidium*-derived compounds and reinforce the genus as a valuable source of structurally novel natural products with biomedical significance.

In this study, we report the isolation and comprehensive characterization of *Phormidium ambiguum*, a thermotolerant filamentous cyanobacterium collected from the Chundzha thermal springs in southeastern Kazakhstan. This research integrates morphological, physiological, and biochemical analyses aimed at elucidating the adaptive and metabolic features of this non-model strain, with particular emphasis on its pigment composition and secondary metabolite profile. The immunomodulatory properties of *P. ambiguum* extracts were evaluated in vitro using three staining panels for T cells, antigen-presenting cells, and natural killer (NK) cells. Human peripheral blood mononuclear cells (PBMCs) from healthy donors were used to determine the activation status and composition of major immune cell subsets by flow cytometry, allowing for direct assessment of the immunostimulatory effects of cyanobacterial metabolites. In addition, the antioxidant and antibacterial activities of the extracts were examined, revealing the multifunctional biological potential of *P. ambiguum*. Unlike well-established model species, thermophilic filamentous cyanobacteria remain underexplored despite their ecological resilience and the distinctive metabolic traits shaped by their extreme habitats. This study therefore addresses a clear knowledge gap by generating foundational biological and analytical data for *P. ambiguum*.

## 2. Results

### 2.1. Characterisation of New Isolated Thermotolerant Phormidium ambiguum Strain

*Phormidium ambiguum* is a filamentous, non-heterocystous cyanobacterium that lacks specialized nitrogen-fixing cells (heterocytes). The trichomes are unbranched, generally straight, and elongated, occasionally forming gentle curves or spiral arrangements when aggregated. The filaments possess a smooth surface and are surrounded by a thin, compact mucilaginous sheath, exhibiting slow gliding motility characteristic of the genus. The cells are cylindrical, with a length slightly exceeding their width (1.84 ± 0.2 µm), and contain finely granular cytoplasm with a green to cyan-green coloration. Akinetes were not observed, and the culture color changed from light green during early growth to olive-green or yellowish-olive in later stages.

**Figure 1:**
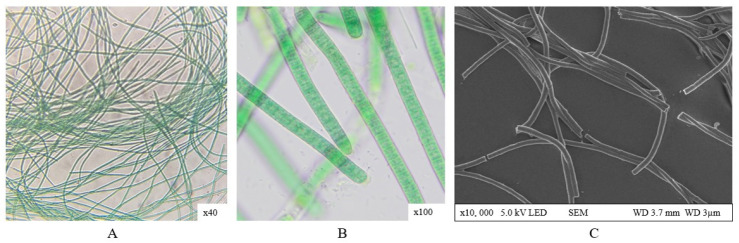
Morphological characteristics of *P. ambiguum* strain: (**A**) Light microscopy (×40); (**B**) close-up of trichomes (×100); (**C**) SEM image showing filament structure (×10,000).

The observed morphological characteristics of the isolate correspond well to the diagnostic features described for representatives of the genus *Phormidium* (order *Oscillatoriales*). Members of this genus are characterized by unbranched, motile trichomes enclosed in thin or indistinct sheaths, the absence of heterocytes and akinetes, and cylindrical cells whose length slightly exceeds their width [39]. The morphology of the isolated strain, including its straight and unbranched trichomes, smooth filament surface, compact mucilaginous sheath, and lack of nitrogen-fixing structures, is consistent with descriptions of *P. ambiguum* provided in previous taxonomic studies [40,41]. Similar features were reported for *P. ambiguum* strains isolated from thermal habitats and soil crusts, where cells typically measure between 1.5 and 2.5 µm in diameter and exhibit gliding motility without visible constrictions at the cross walls [42]. The combination of these traits, together with the absence of gas vesicles and the characteristic olive-green pigmentation, supports the identification of the newly isolated strain as *P. ambiguum*. Such morphological congruence with reference descriptions suggests that the strain maintains typical structural adaptations of thermophilic *Phormidium* species, enabling survival and stability under fluctuating temperature and light conditions (Figure 1).

**Figure 2:**
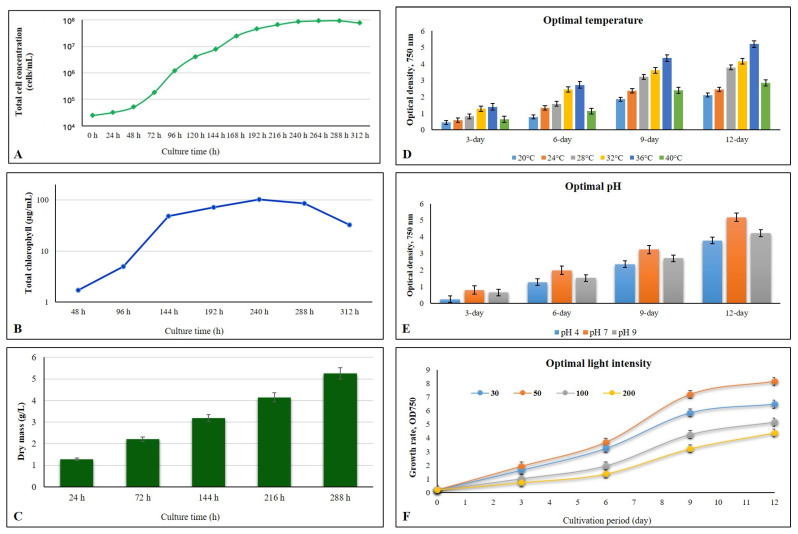
Growth dynamics and optimization of cultivation parameters for *P. ambiguum*: (**A**) cyanobacterial growth rate illustrated by cell numbers; (**B**) cyanobacterial growth rate illustrated by chlorophyll measurements; (**C**) cyanobacterial growth rate illustrated by dry mass; (**D**) determination of optimal temperature for cyanobacterial growth; (**E**) determination of optimal pH for cyanobacterial growth; (**F**) determination of optimal light intensity for cyanobacterial growth.

Furthermore, the isolated *P. ambiguum* strain demonstrated rapid growth and strong environmental resilience, making it suitable for large-scale cultivation. The strain grew actively under moderate illumination (≈50 µmol m^−2^ s^−1^) and temperature conditions of 34–36 °C, achieving optimal biomass accumulation (5.26 g L^−1^) by the 12th day of cultivation (Figure 2). Growth was favored at neutral pH 7 and remained stable across a broad salinity range of 15–60 ppt, with the highest productivity observed near 40 ppt. During cultivation, the culture color changed from light green to oil-green and finally to yellowish-olive at late growth stages, indicating high pigment accumulation. When left undisturbed, the culture showed rapid spontaneous sedimentation of filaments within approximately 2.5 h, forming a compact biomass pellet and a clear supernatant. This feature, together with the ability to separate biomass efficiently by filtration through a 50–100 µm plankton net, represents a major advantage for downstream processing. Additionally, after storage and thawing, the culture released an intense blue coloration associated with the water-soluble pigment phycocyanin—a compound of recognized industrial value as a natural antioxidant and colorant.

**Figure 3:**
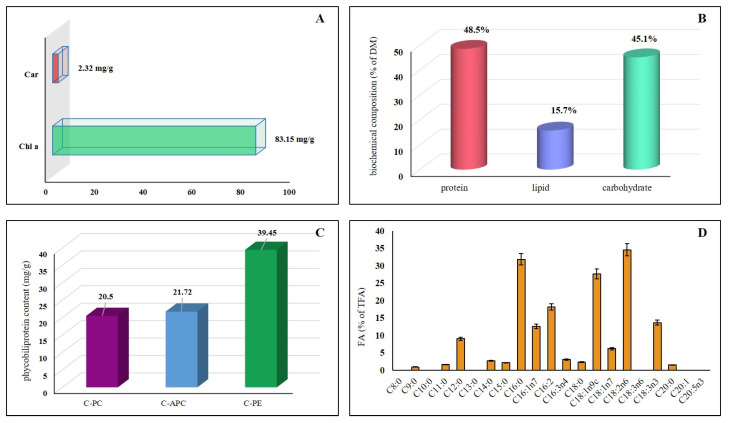
Biochemical profile of *P. ambiguum:* (**A**) chlorophyll *a* and total carotenoids content; (**B**) Total protein, lipid, and carbohydrate content in dry biomass; (**C**) Phycobiliprotein content, including C-phycocyanin (C-PC), allophycocyanin (C-APC), and phycoerythrin (C-PE); (**D**) Fatty acid profile expressed as percentage of total fatty acids (TFA).

*P. ambiguum* demonstrated a balanced biochemical composition characterized by high protein (48.5% DM) and moderate lipid (15.7% DM) contents (Figure 3B). Fatty acid profiling revealed the presence of 11 components, among which palmitic acid (C16:0, 31.89%) and linoleic acid (C18:2, 34.61%) were dominant. Saturated fatty acids (SFAs) accounted for 36.4% of the total fraction, whereas polyunsaturated fatty acids (PUFAs) represented 45.5%, indicating a favorable profile for nutritional and biotechnological applications (Figure 3D). Despite the moderate lipid accumulation, the strain exhibited a high carbohydrate content of 45.1% DM, reflecting its potential for biofuel or feedstock production. As expected for cyanobacteria, chlorophyll *a* constituted approximately 90% of the total photosynthetic pigment pool, with a content of 83.15 µg/mL, while no chlorophyll *b* was detected. Carotenoids were measured at 2.32 µg/mL, and the extract contained echinenone, zeaxanthin, and small amounts of myxoxanthophylls (Figure 3A).

The biochemical profile of *P. ambiguum* demonstrates several similarities to other thermophilic cyanobacteria known for their high-value bioproducts. Comparable to *Thermosynechococcus elongatus* and *Leptolyngbya* sp., the strain exhibited a high protein fraction and a notable accumulation of carbohydrates, confirming its suitability as a potential feed or fertilizer supplement under high-temperature conditions [43,44]. The lipid content and fatty acid distribution were also consistent with other thermotolerant *Phormidium* isolates, which typically contain elevated levels of palmitic and linoleic acids—fatty acids that contribute to membrane stability and thermotolerance [19]. The dominance of chlorophyll a and carotenoids such as zeaxanthin and echinenone further supports the role of *P. ambiguum* as a producer of pigments with antioxidant and immunoprotective properties, as previously reported for *Phormidium valderianum* and *Phormidium corium* [45,46].

In addition, representatives of the genus *Phormidium* have been recognized for their ability to synthesize isoprenoids and phycocyanin in quantities comparable to industrially exploited strains of *Arthrospira* [47]. The present findings confirm that *P. ambiguum* combines several advantageous traits—rapid growth, high protein and carbohydrate yields, and valuable pigment production—making it a promising candidate for sustainable biomass generation and the development of bioactive formulations adapted to thermophilic cultivation systems.

### 2.2. Immunomodulatory Properties of P. ambiguum Extract

To investigate the immunomodulatory potential of *P. ambiguum*, flow cytometric analysis was performed on human peripheral blood mononuclear cells (PBMCs) treated in vitro with various concentrations of the cyanobacterial extract (10–60 µM). The staining panels included major surface markers that define T cells (CD3, CD4, CD8, CD25, CD152), B cells (CD19), antigen-presenting cells (HLA-DR-DP), NK cells (CD16/CD56), and monocytes/macrophages (CD11b). The expression of these markers was compared between extract-treated and untreated cells to assess changes in immune activation and subset distribution. Following in vitro treatment with the *P. ambiguum* extract, a significant activation of T lymphocytes was observed, as reflected by increased percentages of CD3^+^, CD4^+^, CD25^+^, and CD152^+^ cells compared with untreated controls (Table 1).

A significant increase in the proportion of CD3^+^ (T) cells was detected after treatment with *P. ambiguum* extract (19.6 ± 3.1%) compared with untreated controls (11.9 ± 4.4%). Among these, CD4^+^ helper T cells exhibited a marked elevation (16.8 ± 2.4%) relative to control (10.5 ± 3.5%), while CD8^+^ cytotoxic T cells showed a moderate but consistent rise (2.0 ± 1.1%). Furthermore, a distinct increase was observed in the activated T-cell populations expressing CD25^+^ and CD152^+^ markers (4.9 ± 1.3% and 14.2 ± 2.0%, respectively), indicating enhanced activation and regulatory potential.

Notably, *P. ambiguum* extract also upregulated HLA-DR-DP expression in antigen-presenting cells (10.2 ± 1.5% vs. 4.3 ± 1.8% in controls), suggesting a stimulation of adaptive immune signaling pathways. In contrast, no significant changes were detected in the percentages of CD19^+^ B cells, CD11b^+^ macrophages, or CD16^+^CD56^+^ NK cells, indicating that the primary immunomodulatory action of the extract is directed toward T-cell activation and antigen presentation rather than general leukocyte proliferation.

**Figure 4:**
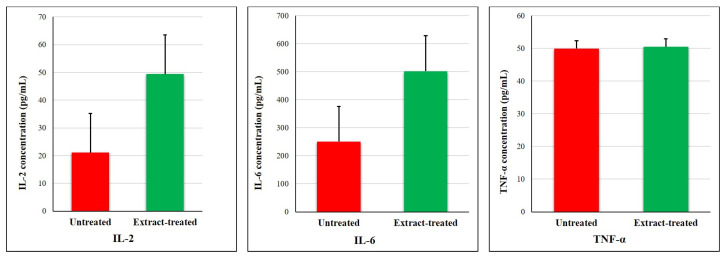
Effect of *P. ambiguum* extract on cytokine secretion. Levels of IL-2, IL-6, and TNF-α were determined in culture supernatants by ELISA. Data are expressed as mean ± SD (n = 5).

Furthermore, cytokine quantification confirmed the stimulatory effects of *P. ambiguum* metabolites on immune function. Treatment resulted in an increased secretion of interleukin-2 (IL-2) and interleukin-6 (IL-6) by 2.3-fold and 2.1-fold, respectively, compared with untreated cells (Figure 4). No significant alteration was detected in tumor necrosis factor-α (TNF-α) levels, indicating a selective activation pattern that favors T-cell proliferation and immune regulation without triggering strong proinflammatory responses.

The immunophenotyping results demonstrate that *P. ambiguum* extract exerts a selective and biologically meaningful stimulatory effect on human immune cells, predominantly enhancing T-cell activation. The extract-induced expansion of total CD3^+^ T cells, with a clear bias toward CD4^+^ helper T cells and an accompanying rise in CD25^+^ and CD152^+^ activation/regulatory markers, indicates that the bioactive compounds present in the extract promote both effector and regulatory components of adaptive immunity. The concurrent upregulation of HLA-DR/DP on antigen-presenting cells further supports the notion that the extract enhances antigen-presentation pathways, which are critical for initiating T-cell–mediated responses. Importantly, the absence of significant changes in NK cells, B cells, and monocyte/macrophage populations suggests that the extract does not induce broad leukocyte activation, but instead modulates specific immune pathways.

Comparable immune-modulating activity has been described for *Phormidium papyraceum*, which directed PBMCs toward a CD4^+^ effector/memory phenotype and markedly increased IL-2 (55 ± 12 pg/mL) and IL-6 (493 ± 64 pg/mL) secretion while leaving TNF-α unchanged [48]. This controlled pattern of cytokine release—enhanced IL-2 and IL-6 without excessive TNF-α—is nearly identical to the cytokine profile induced by *P. ambiguum* in our study, suggesting that both strains activate T-cells in a targeted and physiologically balanced manner.

Additional insight comes from studies on *Spirulina platensis*, particularly those investigating C-phycocyanin (C-PC), which plays a role in modulating autoimmune and neuroinflammatory processes. In an experimental autoimmune encephalitis (EAE) model, C-PC decreased disease severity and upregulated key markers of regulatory T cells—FOXP3, CD25, IL-10, and TGF-β—while simultaneously exerting neuroprotective effects that mitigated myelin and axonal damage [49]. These findings broaden the understanding of cyanobacterial metabolites as modulators of T-cell differentiation and underscore the relevance of pigment–protein complexes that may underlie the immunoregulatory activity observed in *P. ambiguum*.

Recent report on *Cyanobacterium* sp. Rippka B-1200, where both endo- and exopolysaccharide fractions demonstrated clear immunomodulatory potential in vivo. Polysaccharides with molecular weights of 6–8 kDa produced dose-dependent immune effects: while endopolysaccharides at 300 mg/kg did not significantly alter thymus index in immunosuppressed mice (1.60 ± 0.15 mg/g vs. 1.15 ± 0.24 mg/g), exopolysaccharides at 600 mg/kg restored thymus mass (3.60 ± 0.32 mg/g) to levels comparable to non-immunosuppressed controls (3.70 ± 0.25 mg/g). At the same dose, endopolysaccharides also exhibited strong immunostimulatory activity without inducing pathological changes in internal organs [50]. These results highlight the capacity of cyanobacterial polysaccharides to reverse chemically induced immunosuppression—an effect conceptually aligned with the selective T-cell activation and cytokine recovery observed for *P. ambiguum*.

### 2.3. Antioxidant Capacity of P. ambiguum Extract

**Figure 5:**
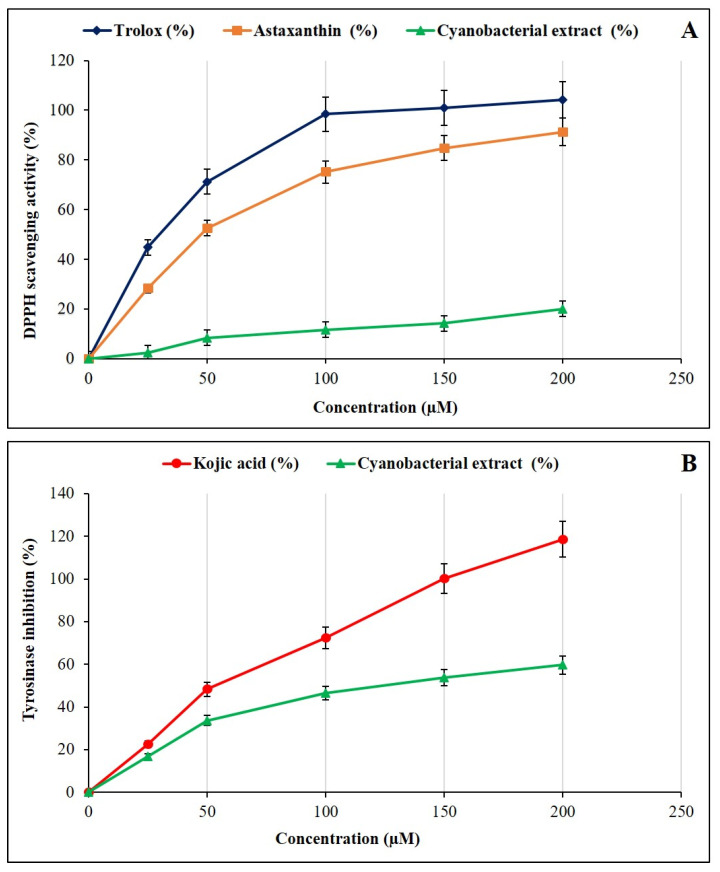
(**A**) The DPPH (2,2-diphenyl-1-picrylhydrazyl) free radical scavenging activity of the *P. ambiguum* extract in comparison with standard antioxidants. Trolox and astaxanthin were used as positive controls. (**B**) Inhibitory effect of the *P. ambiguum* extract on tyrosinase activity using L-DOPA as a substrate. Kojic acid was used as a positive control. The data represent mean ± SD, *p* < 0.01.

The antioxidant properties of the *P. ambiguum* extract were assessed by the DPPH radical scavenging assay, and the results revealed a moderate, dose-dependent increase in activity (Figure 5A). At the highest tested concentration (200 μM), the extract achieved 20.1% scavenging activity, which was considerably lower than that of the reference antioxidants Trolox and astaxanthin, reaching 104.2% and 91.3%, respectively. Although the extract displayed a weaker scavenging capacity, its progressive increase across the tested concentration range suggests the presence of metabolites capable of donating electrons or hydrogen atoms to neutralize DPPH radicals. Such moderate radical-scavenging efficiency is typical for cyanobacterial extracts, where the antioxidant effect often arises from a complex mixture of fatty acids, phenolic derivatives, and carotenoid-like pigments rather than a single dominant compound. These findings indicate that *P. ambiguum* contains biologically active molecules with potential antioxidant properties, though their overall contribution may depend on their concentration and polarity within the crude extract.

In the tyrosinase-inhibition assay (Figure 5B), the *P. ambiguum* extract also demonstrated measurable inhibitory activity against the fungal tyrosinase enzyme, showing a dose-dependent response between 25 and 200 μM. At the maximum tested concentration, the extract inhibited tyrosinase activity by approximately 60%, compared to the complete inhibition observed with the positive control, kojic acid. Tyrosinase is a copper-containing enzyme that catalyzes the initial steps of melanin biosynthesis, and its inhibition is widely explored in cosmetic and pharmaceutical applications for its skin-lightening and anti-browning effects. The observed inhibition by *P. ambiguum* therefore suggests that its constituents may interact with the enzyme’s active site or chelate the copper ions required for catalysis. Thus, while the extract does not match the potency of established antioxidants, it exhibits a clear biological effect that warrants further molecular investigation.

When compared with other cyanobacteria, the antioxidant profile of *P. ambiguum* aligns with reports describing moderate activities in non-optimized crude extracts. For instance, methanolic extracts of *Nostoc*, *Oscillatoria*, and other filamentous strains frequently exhibit DPPH inhibition rates of between 50–80% at higher concentrations, contingent on pigment and phenolic content [51]. Extracts from *Phormidium autumnale* also demonstrated relatively high radical-scavenging activity, which has been attributed to the abundance of phycobiliproteins and carotenoids such as zeaxanthin, canthaxanthin, echinenone, and myxoxanthophyll derivatives [52]. Furthermore, optimised extractions from *Spirulina* (*Arthrospira*) species frequently surpass 70% DPPH inhibition and correlate strongly with C-phycocyanin concentration, total phenolics, and polysaccharide-associated antioxidant mechanisms [53].

In contrast, the present study utilised a non-polar extraction protocol, a method that selectively enriches lipophilic compounds while excluding phycobiliproteins, which are key contributors to antioxidant performance in many cyanobacteria. The moderate activity observed in this study is therefore consistent with the chemical nature of the extracted fraction. UHPLC-MS analysis confirmed a diverse mixture of fatty-acid derivatives, terpenoids, carotenoid-like molecules, and low-abundance pigments. It is evident that a significant proportion of these compound classes have been demonstrated to exert antioxidant or radical-quenching effects, albeit typically to a lesser extent than the protein-bound or water-soluble antioxidants that are predominantly present in polar fractions.

Taken together, the results suggest that the antioxidant potential of *P. ambiguum* is meaningful but not maximal in its current extraction form. Given the strain’s thermophilic origin and metabolic diversity, it is plausible that stronger antioxidant activity could be obtained through (i) fractionation-guided purification, (ii) inclusion of polar pigment-rich extracts, or (iii) cultivation under stress conditions known to enhance carotenoid and phenolic biosynthesis. It is important to note that the moderate antioxidant activity should not be viewed in isolation: in this strain, antioxidant, immunomodulatory, and antibacterial properties co-occur, indicating that *P. ambiguum* may have a broader biological profile than species optimized exclusively for redox activity.

### 2.4. Antibacterial Potential of P. ambiguum Extract

The antibacterial activity of the *P. ambiguum* extract was assessed using the agar diffusion method to determine its inhibitory effect against both Gram-negative and Gram-positive bacteria. Six bacterial strains were selected, including three Gram-negative species (*Escherichia coli*, *Pseudomonas aeruginosa*, and *Proteus vulgaris*) and three Gram-positive species (*Bacillus subtilis*, *Micrococcus luteus* ATCC 49732, and *Staphylococcus epidermidis* ATCC 49134). Penicillin/streptomycin was used as a positive control, while DMSO/water (1:1) served as a negative control. The results demonstrated measurable inhibition zones for all tested bacteria, confirming the antimicrobial efficacy of the cyanobacterial extract (Table 2).

The inhibition zones of *P. ambiguum* extract ranged from 9.85 ± 0.72 mm to 18.65 ± 1.24 mm, indicating moderate but consistent antibacterial activity. Among Gram-negative strains, the highest inhibition was observed against *E. coli* (18.65 ± 1.24 mm), followed by *P. vulgaris* (17.42 ± 1.10 mm) and *P. aeruginosa* (15.78 ± 0.96 mm). For Gram-positive bacteria, inhibition zones ranged between 9.85 ± 0.72 mm (*S. epidermidis*) and 14.68 ± 0.83 mm (*B. subtilis*), with *M. luteus* showing intermediate sensitivity (12.47 ± 0.91 mm). As expected, antibiotics produced significantly larger inhibition zones (24.2–27.5 mm), whereas no inhibition was detected for the DMSO control.

Overall, *E. coli* was identified as the most sensitive strain to the *P. ambiguum* extract, while S. epidermidis exhibited the highest resistance. The greater susceptibility of Gram-negative bacteria may be attributed to differences in cell wall composition and permeability, allowing for easier penetration of lipophilic compounds from the cyanobacterial extract. The antibacterial activity observed here supports previous findings on *Leptolyngbya* species, whose extracts have shown bacteriostatic effects due to the presence of bioactive metabolites such as fatty acids, alkaloids, and phenolic pigments [54]. Furthermore, the ethanol extracts of *Phormidium* sp. and *Microcoleus* sp. at various concentrations (0.2, 0.06, 0.03 and 0.015 g/mL) showed the antibacterial activity against *Streptococcus enteritidis* and *E. coli* on the media [55]. These compounds are known to disrupt microbial membrane integrity and inhibit enzymatic processes essential for bacterial survival.

### 2.5. Biochemical Composition of P. ambiguum Extract

**Figure 6:**
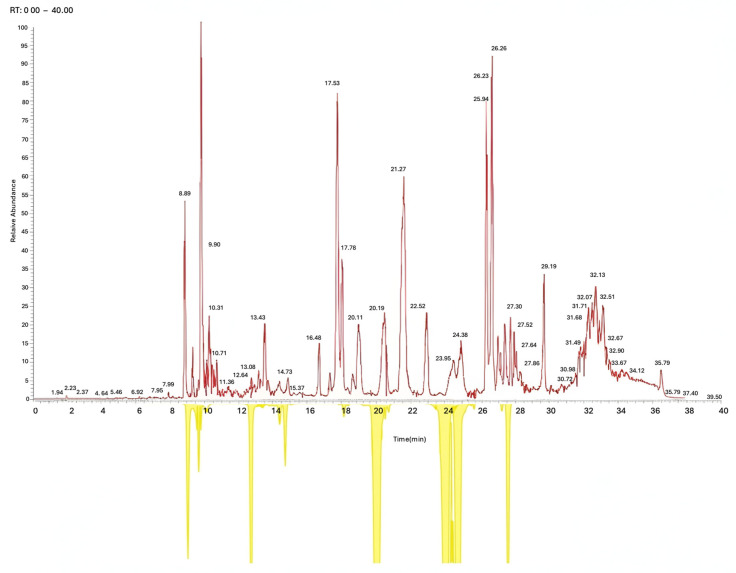
LC–ESI–MS chromatographic profile of the non-polar extract of *P. ambiguum* acquired in positive ion mode using a Q-TOF mass spectrometer.

The LC-ESI-MS/MS analysis of the non-polar fraction of the *P. ambiguum* extract revealed the presence of a chemically diverse set of metabolites, with more than 1000 distinct molecular features detected across the chromatographic run. The complete chromatographic profile is shown in Figure 6. The upper trace (red) represents the total ion current (TIC) chromatogram recorded in the mass range of 10–800 *m*/*z*, providing an overview of all ionizable compounds eluting from 0 to 40 min. The lower trace (yellow) highlights extracted-ion chromatographic features corresponding to putative metabolites listed in Table 3. Both traces were autoscaled to the maximum signal intensity to facilitate comparison of peak distribution and relative abundance.

To guide interpretation of this profile, it is important to note that the data presented were acquired exclusively in positive ESI mode. Preliminary analyses performed in both positive and negative ionization modes demonstrated that the lipophilic extract—rich in fatty acid derivatives, terpenoids, sterols, and pigment-related molecules, ionized far more efficiently under positive-ion conditions. In contrast, spectra obtained in negative mode contained only weak and sparse ion signals and did not provide additional structural information beyond that captured in positive mode. This behavior is consistent with the chemical nature of the fraction analyzed, as non-polar metabolites typically show higher ionization efficiency and improved fragmentation patterns in positive ESI.

The TIC reveals a highly complex chemical matrix typical of cyanobacterial lipid-rich extracts. Numerous well-resolved peaks indicate the presence of multiple metabolite classes, with the majority eluting between 10 and 35 min. Early-eluting peaks (RT 0–10 min) likely correspond to low-molecular-weight or moderately polar constituents, whereas the dense region from 17 to 30 min reflects the elution of more hydrophobic compounds. Several dominant peaks (RT 17.5, 26.0–26.3, and 32 min) suggest the presence of abundant long-chain fatty acid derivatives, terpenoid alcohols such as phytol, amides of fatty acids, and sterol- or carotenoid-related structures. These classes of metabolites commonly exhibit strong ionization in the positive electrospray mode, consistent with the intensity patterns observed in the TIC.

The later retention window (RT 30–36 min) contains peaks characteristic of highly hydrophobic molecules, including carotenoid derivatives and sterols, which are frequently reported in lipid extracts of filamentous cyanobacteria. The distribution and intensity of these peaks underscore the metabolic richness of *P. ambiguum*, supporting the LC-MS/MS identification of multiple lipid-associated components, pigments, and terpenoid derivatives.

From revealed compounds, 20 metabolites were selected for detailed annotation based on signal quality, isotopic pattern, MS/MS fragmentation and reported biological relevance (Table 3). For each compound, the ion type, calculated and experimentally observed *m*/*z* values, and mass error (ppm) are provided. The dataset includes several fatty acid amides (palmitic amide, oleamide), glycolipids (MGDG and DGDG 16:2/18:2), terpenoids and carotenoids (zeaxanthin, β-carotene, echinenone), as well as chlorophyll a and other secondary metabolites such as oscillatoxin A, oscillamid Y and betulin. Carotenoids and chlorophyll a were mainly detected as radical cations (M•^+^), whereas amides, glycolipids and macrolides appeared predominantly as protonated molecular ions [M + H]^+^, which is consistent with their expected ionization behaviour in positive ESI mode.

The analysis identified several key groups of metabolites, including fatty acids and their derivatives, carbamate and carboxylate esters, terpenoids, amides, secondary and tertiary alcohols, as well as photosynthetic pigments such as carotenoids and phycocyanin. Many of these metabolites have been previously associated with biological activities such as antibacterial, antioxidant, antiviral, antifungal, and cytotoxic effects, as well as enzyme inhibition. Among the predominant compounds, linoleic acid, palmitic acid methyl ester, phytol, hexadecanamide, and β-sitosterol were the most abundant, reflecting a biochemical composition comparable to other bioactive cyanobacterial extracts [56,57,58].

The presence of these compound classes is further contextualized by studies on other cyanobacteria, which have been shown to produce structurally diverse lipids, peptides, and pigment-derived metabolites with notable anti-inflammatory and immunomodulatory properties observed in this study. For example, *Spirulina* species are known to accumulate sulphoquinovosyl diacylglycerol (SQDG) and other glycolipids such as monogalactosyldiacylglycerides (MGDGs) and glycosphingolipids, together with phospholipids including phosphatidylcholine (PC) and phosphatidylethanolamine (PE). In *Spirulina subsalsa*, glycolipid- and phospholipid-rich fractions demonstrated potent anti-inflammatory effects, indicating their potential use in developing cardiovascular and immune-support formulations [59]. Bioactive peptides isolated from *Synechococcus* sp. (<3 kDa) likewise showed pronounced anti-inflammatory activity by reducing the gene expression of key mediators such as iNOS, TNF-α, COX-2, and IL-6 in LPS-stimulated RAW 264.7 macrophages, suggesting their potential for the development of natural anti-inflammatory therapeutics [10]. A recent study on a *Leptolyngbya*-like strain (LEGE 13412) reported a reduction in nitric oxide production in macrophages, highlighting its possible relevance to psoriasis treatment [60]. Similarly, lipophilic fractions from *Gloeothece* sp. inhibited COX-2 by approximately 58% at low concentrations (10 µg mL^−1^), demonstrating potent anti-inflammatory activity associated with polar lipid constituents [61].

Additional examples of cyanobacterial metabolites with strong immunomodulatory potential include the compound (9S,E)-8-ethyl-9-methylnonadec-6-en-3-one (EME) from *Lyngbya* sp., which downregulated COX-2, TNF-α, iNOS, NF-κB, and IL-1β expression in LPS-stimulated macrophages [62], and Unnarmicin D from *Trichodesmium thiebautii*, which reduced TNF-α, IL-16, soluble TLR2, and nitric oxide levels, identifying it as a promising scaffold for neuroinflammation-targeted therapies [63]. Malynglamide F—another compound isolated from marine *Lyngbya*—was shown to decrease IL-6 and TNF-α in multiple inflammation-induced rat models, further demonstrating the strong therapeutic potential of cyanobacterial metabolites in inflammatory disorders [64].

Collectively, these examples underscore the biochemical richness of cyanobacteria and highlight the broad spectrum of biological activities contributed by their lipid-, peptide-, and pigment-derived metabolites. In this context, the diverse metabolite composition detected in *P. ambiguum* is consistent with the metabolic capabilities observed in other cyanobacterial taxa, reinforcing the view that cyanobacteria represent a valuable reservoir of natural compounds with immunomodulatory, antioxidant, and anti-inflammatory potential. The presence of multiple bioactive classes within a single extract suggests that the biological effects observed for *P. ambiguum* may arise from combinatorial or synergistic interactions among its metabolites, warranting further fractionation-guided studies to identify the key molecular drivers of activity.

## 3. Materials and Methods

### 3.1. Biomass Production

A thermotolerant cyanobacterial strain identified as *Phormidium ambiguum* was isolated from hot springs located in the Chundzha settlement of the Uygur district, Almaty region, Kazakhstan (43°47′ N, 79°14′ E), approximately 250 km southeast of Almaty city. Water and biofilm samples were collected aseptically into sterile 50 mL Falcon tubes, transported at 4 °C, and processed within 24 h. Enrichment cultures were established using standard microbiological techniques, including serial dilution, repeated reseeding, and single-filament isolation using a micromanipulator under a stereomicroscope. To obtain an axenic culture, the isolate was repeatedly subcultured on solid BG-11 agar supplemented with cycloheximide (50 µg mL^−1^) and ampicillin (10 µg mL^−1^) to inhibit fungal and bacterial contaminants. Morphological identification was carried out using a Leica DM750 light microscopy (Leica Microsystems, Wetzlar, Germany) and scanning electron microscopy (Auriga Crossbeam 540, Cari Zeiss Microscopy GmbH, Oberkochen, Germany). Cell density and growth dynamics were monitored spectrophotometrically at 750 nm (UV-1800, Shimadzu Corporation, Kyoto, Japan). Chlorophyll a concentration was determined according to Bennet and Bogorad [65] with absorbance readings at 652 and 665 nm.

Axenic cultures of *P. ambiguum* were maintained in 500 mL Erlenmeyer flasks containing 350 mL of liquid medium. Growth experiments were performed in two parallel nutrient systems: (i) a modified Zarrouk medium (g L^−1^: NaCl 1.0, CaCl_2_·2H_2_O 0.04, KNO_3_ 2.5, FeSO_4_·7H_2_O 0.01, Na_2_EDTA 0.08, K_2_SO_4_ 1.0, MgSO_4_·7H_2_O 0.2, NaHCO_3_ 16.8, K_2_HPO_4_ 0.5) [66], and (ii) BG-11 medium (g L^−1^: NaNO_3_ 1.5, CaCl_2_·2H_2_O 0.036, ferric ammonium citrate 0.012, Na_2_EDTA·2H_2_O 0.001, K_2_HPO_4_ 0.04, MgSO_4_·7H_2_O 0.075, Na_2_CO_3_ 0.02, and 1 mL of trace metals solution containing H_3_BO_3_ 2.86, MnCl_2_·4H_2_O 1.81, ZnSO_4_·7H_2_O 0.222, Na_2_MoO_4_·2H_2_O 0.39, CuSO_4_·5H_2_O 0.079, and Co(NO_3_)_2_·6H_2_O 0.049) [67].

Cultivation was conducted under controlled laboratory conditions using a thermostatic photobioreactor (Biostat A+, Sartorius Stedim Biotech, Göttingen, Germany) at 34–36 °C with continuous aeration (1 L min^−1^) by a sterile air/CO_2_ mixture (1% *v*/*v*). The cultures were illuminated at 50 µmol photons m^−2^ s^−1^ using white LED lamps under a 12:12 h light–dark cycle. Growth was monitored daily by measuring optical density and dry weight. Cultures reached the stationary phase after approximately 12 days. At harvest, biomass was separated by centrifugation at 4500 rpm for 10 min using a refrigerated centrifuge (Eppendorf 5804R, Hamburg, Germany). The resulting pellets were washed twice with sterile distilled water, frozen at −80 °C, and lyophilized (Christ Alpha 1–4 LDplus, Martin Christ Gefriertrocknungsanlagen GmbH, Osterode am Harz, Germany). The dry biomass was stored at −20 °C until further biochemical and bioactivity analyses.

### 3.2. Extract Preparation

Lyophilized biomass of *P. ambiguum* (500 mg dry weight) was used for metabolite extraction. The sample was suspended in 3 mL of analytical-grade methanol (MeOH; Sigma-Aldrich, St. Louis, MO, USA) and vortexed for 1 min to achieve homogenization. The mixture was then subjected to ultrasonic-assisted extraction in an ultrasonic bath (Branson 2800, Branson Ultrasonics, Danbury, CT, USA) for 20 min at 25 °C with intermittent vortexing to enhance solvent penetration and metabolite release. Following sonication, 6 mL of chloroform were added to the suspension, and the mixture was agitated on an orbital shaker (IKA KS 130, IKA-Werke GmbH & Co. KG, Staufen, Germany) for 20 min at 150 rpm to ensure complete extraction of non-polar and semi-polar compounds. Subsequently, 3 mL of Milli-Q ultrapure water were added to induce phase separation, and the mixture was vortexed for 1 min.

The biphasic extract was centrifuged at 4000 rpm for 20 min at 4 °C using a refrigerated centrifuge (Eppendorf 5804 R, Hamburg, Germany). The lower methanol/chloroform layer containing the non-polar fraction was carefully collected and filtered through a 0.20 µm hydrophobic PTFE syringe filter (Millex-FG, Merck KGaA, Darmstadt, Germany) to remove residual particulates. The solvent was then evaporated under reduced pressure at 38 °C using a rotary evaporator (Büchi R-210, Büchi Labortechnik AG, Flawil, Switzerland) until complete dryness.

The obtained dry extract was re-dissolved in 50% (*v*/*v*) aqueous dimethyl sulfoxide (DMSO) to a final concentration of 5 mg mL^−1^ (5 mg of residue per 1 mL of DMSO/water = 1:1) and stored at 4 °C in amber glass vials until further use. Working solutions were freshly prepared by diluting the stock solution in Dulbecco’s phosphate-buffered saline (DPBS; Gibco^®^, Life Technologies™, Paisley, UK) to the required concentrations for each biological assay.

### 3.3. Immunological Assay

Peripheral blood mononuclear cells (PBMCs) were isolated from venous blood samples collected from healthy adult volunteers (n = 10) using density-gradient centrifugation with Ficoll-Paque™ PLUS (GE Healthcare, Uppsala, Sweden). This study was conducted in accordance with institutional ethical standards and with informed consent from all donors. The obtained leukocyte fraction was washed twice in sterile PBS (pH 7.4) and centrifuged at 1500× *g* for 10 min at room temperature. The resulting cell pellet was resuspended in Dulbecco’s Modified Eagle’s Medium (DMEM; Sigma-Aldrich Chemie GmbH, Steinheim, Germany) supplemented with 10% heat-inactivated fetal bovine serum (FBS; Gibco^®^, Thermo Fisher Scientific, Waltham, MA, USA), 100 U/mL penicillin, 100 μg/mL streptomycin, and 0.25 μg/mL amphotericin B. This formulation was designated as complete DMEM.

Cells were seeded in 12-well TPP (Trasadingen, Switzerland) at a density of 1 × 10^6^ cells/mL (1 mL per well) and incubated at 37 °C in a humidified atmosphere containing 5% CO_2_. After 24 h of stabilization, cultures were treated with 4 μL of *P. ambiguum* extract stock solution (5 mg/mL in DMSO/water, 1:1) to achieve a final concentration of 20 μg/mL in the medium. The exposure period was 48 h. Untreated cells maintained in complete DMEM served as the negative control, while a DMSO/water (1:1) vehicle control (4 μL/mL) was used to evaluate solvent effects. Cells incubated with 1 μg/mL PHA-L (Sigma-Aldrich, St. Louis, MO, USA) were used as a positive control for mitogenic activation.

Pilot cytotoxicity assays performed on tumor cell lines: MiaPaCa2 (pancreatic carcinoma), HepG2 (hepatocellular carcinoma), and K562 (chronic myelogenous leukemia) confirmed that *P. ambiguum* extract concentrations up to 100 μg/mL exhibited no cytotoxic effects after 48 h of incubation, validating the use of 20 μg/mL for immunomodulatory evaluation.

After treatment, cells were harvested and subjected to immunophenotypic analysis by flow cytometry using a Cytomics FC500 system (Beckman Coulter Life Sciences, Indianapolis, IN, USA) operated with CXP acquisition and analysis software (version 2.2, Beckman Coulter Life Sciences). Three staining panels were applied to assess T lymphocytes (CD3, CD4, CD8, CD25), antigen-presenting cells (HLA-DR, HLA-DP, CD14), and natural killer (NK) cells (CD16, CD56, CD69). Fluorescently conjugated monoclonal antibodies were obtained from BD Biosciences (San Jose, CA, USA). Data were analyzed using CytExpert 2.4 software (Beckman Coulter).

This study was conducted in accordance with the ethical principles outlined in the Declaration of Helsinki and approved by the Local Ethics Committee of Al-Farabi Kazakh National University (Almaty, Kazakhstan; Protocol No. IRB-A694, approved on 11 September 2024). Written informed consent was obtained from all participants prior to sample collection and experimental procedures.

### 3.4. Antioxidant Activity

The antioxidant capacity of the *Phormidium ambiguum* extract was evaluated using the 2,2-diphenyl-1-picrylhydrazyl (DPPH) radical scavenging assay, which is based on the ability of antioxidants to quench the stable DPPH radical through hydrogen or electron donation. The assay was performed following the method of Brand-Williams et al. [68] with minor modifications to optimize sensitivity for cyanobacterial extracts.

Briefly, a 0.1 mM DPPH solution was freshly prepared in analytical-grade methanol (Sigma-Aldrich, Steinheim, Germany) and kept protected from light. In a 96-well flat-bottom microplate (Nunc™, Thermo Fisher Scientific, Roskilde, Denmark), 100 µL of the DPPH solution was added to 100 µL of the sample solution prepared at different concentrations (25–200 µg/mL) in methanol containing 0.5% (*v*/*v*) DMSO. The reaction mixtures were gently mixed and incubated for 30 min in the dark at room temperature (22–25 °C). After incubation, the decrease in absorbance was measured at 515 nm using an EnVision™ multimode microplate reader (PerkinElmer, Boston, MA, USA).

Trolox (6-hydroxy-2,5,7,8-tetramethylchroman-2-carboxylic acid; Sigma-Aldrich) and astaxanthin (Sigma-Aldrich) were used as reference antioxidants. A methanol solution containing 0.5% DMSO served as the blank control, while a DPPH-only solution served as the negative control. Each sample was analyzed in triplicate, and the percentage of radical scavenging activity (%RSA) was calculated using the following equation:

(1) $$RSA\;\left(\%\right)=\frac{Ac-As}{Ac}\;\times 100,$$

where A_c_—the absorbance of the DPPH solution without sample; and A_s_—the absorbance of the reaction mixture with the sample extract.

The tyrosinase-inhibitory activity was assessed spectrophotometrically using fungal tyrosinase (2138 U/mL, Sigma-Aldrich, Steinheim, Germany) and levodopa (L-DOPA; Sigma-Aldrich, Steinheim, Germany) as the substrate, as described by Masuda et al. [21] with slight modifications. In brief, 50 µL of the test extract diluted in phosphate-buffered saline (PBS, pH 6.8) containing 8% (*v*/*v*) DMSO was mixed with 100 µL of L-DOPA solution (1 mg/mL in PBS) and 50 µL of fungal tyrosinase solution (500 U/mL in PBS). The reaction mixture was incubated at 37 °C for 30 min, after which the dopachrome formation was quantified by measuring absorbance at 475 nm using the EnVision reader. Kojic acid (Tokyo Chemical Industry, Oxford, UK) was used as a positive control, while PBS with DMSO served as a negative control. The percentage inhibition of tyrosinase activity was calculated as follows:

(2) $$Inhibition\;(\%)=\left(1-\;\frac{As}{Ac}\;\right)\times 100$$

### 3.5. Antimicrobial Activity

The antibacterial potential of the *P. ambiguum* extract was assessed using the modified agar well diffusion method described by Balouiri et al. [69]. Six bacterial strains were tested: three Gram-negative (*Escherichia coli*, *Pseudomonas aeruginosa*, and *Proteus vulgaris*) and three Gram-positive (*Bacillus subtilis, Micrococcus luteus* ATCC 49732, and *Staphylococcus epidermidis* ATCC 49134*). Each strain was grown overnight in nutrient broth (HiMedia, Mumbai, India) at 37 °C with shaking at 150 rpm. The bacterial suspensions were then adjusted to a turbidity equivalent to 0.5 McFarland standard using a spectrophotometer (UV-1800, Shimadzu Corporation, Kyoto, Japan) at OD_600_. Mueller-Hinton agar (MHA; Oxoid, Hampshire, UK) plates were inoculated uniformly by spreading 50 µL of the standardized bacterial suspension over the surface using sterile cotton swabs. Wells (8 mm) were aseptically punched into the agar using a sterile cork borer, and each well was filled with 20 µL of *P. ambiguum* extract containing 20 µg of dry material. Plates were left at room temperature for 1 h to allow for pre-diffusion and subsequently incubated at 37 °C for 24 h. Streptomycin (10 µg disk) and ampicillin (10 µg disk) (Oxoid, UK) were used as positive antibiotic controls, while DMSO/water (1:1, *v*/*v*) served as the negative control. After incubation, the diameters of inhibition zones were measured in millimeters using a digital Vernier caliper (Mitutoyo 500-196-30, Mitutoyo Corporation, Kawasaki, Japan). All tests were performed in triplicate, and the results were expressed as mean ± standard deviation (SD).

All bacterial cultures were obtained from the Microbial Culture Collection of the Department of Biotechnology, Al-Farabi Kazakh National University, and maintained on nutrient agar slants at 4 °C until use.

### 3.6. LC–MS Analysis

#### 3.6.1. Instrumentation

The qualitative and quantitative profiling of metabolites and pigments in the *P. ambiguum* extract was performed using an UHPLC (Thermo Fisher Scientific, Sunnyvale, CA, USA) equipped with a DAD and coupled to an Impact HD high-resolution Q-TOF mass spectrometer (Bruker Daltonics, Billerica, MA, USA). The instrument was operated under positive electrospray ionization (ESI^+^) mode. Calibration was achieved with sodium formate clusters using an internal LockMass correction. Data acquisition and processing were carried out using Bruker Compass DataAnalysis software (version 4.2) and SmartFormula™ for elemental composition calculation based on accurate mass and isotopic pattern fitting.

#### 3.6.2. Chromatographic Conditions

Chromatographic separation was achieved on a Luna C8(2) 100 Å column (100 mm × 4.6 mm, 3 µm particle size; Phenomenex, Torrance, CA, USA) thermostated at 30 °C. The mobile-phase system consisted of solvent A (80% methanol in deionized water) and solvent B (100% methanol). The gradient elution program was as follows: 0 min, 100% A; 0–20 min, linear increase to 100% B; 20–25 min, 100% B; 25–27 min, return to 100% A; 27–30 min, re-equilibration at 100% A. The flow rate was maintained at 0.8 mL min^−1^, and the injection volume was 20 µL of filtered extract. UV–visible spectra were recorded between 250–700 nm, with specific detection channels at 440 nm and 665 nm for carotenoids and phycobiliproteins, respectively.

#### 3.6.3. Mass Spectrometric Conditions

Mass spectrometric analysis was performed using H-ESI with the following optimized parameters: capillary voltage, 4.0 kV; nebulizer pressure, 2.0 bar; drying gas flow, 8 L min^−1^; and drying temperature, 250 °C. Full-scan MS spectra were acquired in the range *m*/*z* 100–2000 with a resolving power of 70,000 FWHM. MS/MS fragmentation was conducted in DDA mode using stepped collision energies of 20–50 eV. Instrument calibration was verified every 24 h using the manufacturer’s sodium formate standard.

For each detected feature, the ion type—[M + H]^+^, [M + Na]^+^, [M + NH_4_]^+^, or radical cations [M·^+^]—was assigned based on mass accuracy, isotopic pattern, and fragmentation behavior. The identification of carotenoids, fatty acid derivatives, terpenoids, and other metabolites was based on retention time, UV–vis spectral characteristics (λ_max, fine-structure ratio %III/II, and cis-peak intensity %Ab/AII), and accurate mass fragmentation patterns, in comparison with literature data and reference standards.

Internal mass calibration (LockMass correction) was performed using sodium formate cluster ions introduced at the beginning of each LC–MS run, providing reference ions across the *m*/*z* range for real-time mass correction. The sodium formate calibrant generated characteristic cluster ions in positive ESI mode, ensuring mass accuracy within ±5 ppm throughout the analysis.

### 3.7. Statistical Analysis

All experiments were conducted in triplicate, and data are presented as mean ± standard deviation (SD). Prior to statistical testing, the distribution of the acquired data was assessed for normality using the Shapiro–Wilk test, and homogeneity of variances was verified using Levene’s test. For datasets meeting the assumptions of normality and homoscedasticity, statistical differences among multiple groups were evaluated using one-way ANOVA, followed by Tukey’s post hoc test for pairwise comparisons. When normality assumptions were not met, non-parametric alternatives were applied as appropriate. Statistical significance was set at *p* < 0.05, *p* < 0.01, and *p* < 0.001. All analyses were performed using GraphPad Prism (version 8.0).

## 4. Conclusions

In this study, a thermotolerant filamentous cyanobacterium, *P. ambiguum*, was isolated from the Chundzha thermal springs in southeastern Kazakhstan and characterized using morphological, physiological, and biochemical approaches. The strain exhibited rapid growth under laboratory conditions and accumulated substantial levels of proteins, carbohydrates, and photosynthetic pigments, including chlorophyll a, phycocyanin, and zeaxanthin. These features reflect metabolic traits commonly observed in filamentous cyanobacteria adapted to high-temperature environments. Analysis of the non-polar fraction by LC–ESI–MS/MS revealed a chemically diverse metabolite profile, with more than 150 putative lipophilic compounds tentatively identified, mainly comprising fatty acid derivatives, terpenoids, amides, and pigment-associated metabolites previously linked to biological activity.

Functional assays indicated that the *P. ambiguum* extract displays measurable antioxidant, immunomodulatory, and antibacterial effects under controlled in vitro conditions. Specifically, the extract showed moderate DPPH radical-scavenging activity, stimulated activation and proliferation of selected immune cell populations, and inhibited the growth of both Gram-positive and Gram-negative bacteria. These responses are consistent with reported activities of pigment- and lipid-rich cyanobacterial fractions and support the relevance of further mechanistic investigation. At the same time, the observed bioactivities should be interpreted within the experimental framework of this study, as the functional properties of metabolites produced by thermophilic cyanobacteria may be influenced by cultivation parameters and environmental context.

Overall, this study expands the current understanding of thermophilic *Phormidium* species and supports their relevance primarily in the context of exploratory biodiscovery rather than immediate technological application. The bioactivities observed here should be interpreted within the ecological and physiological context of extremophilic cyanobacteria, whose metabolic products may exhibit condition-dependent properties shaped by prolonged exposure to elevated temperatures and specialized environmental pressures. Future investigations should therefore focus on isolating individual metabolites, elucidating their molecular targets, and evaluating their functional stability and biosynthetic regulation under defined and comparative cultivation conditions. Such work will be essential to determine whether metabolites derived from *P. ambiguum* retain their activity beyond their native ecological context and can be realistically considered for further development as antioxidant, antimicrobial, or immunomodulatory agents.

## Figures and Tables

**Table 1:** Immunophenotyping of human leukocytes after in vitro treatment with *P. ambiguum* extract.

CD Markers	Extract-Treated Cells (%)	Untreated Cells (Negative Control, %)	PHA-L Treated Cells (Positive Control, %)
CD3	19.6 ± 3.1 ^a^	11.9 ± 4.4	28.3 ± 3.1
CD4	16.8 ± 2.4 ^a^	10.5 ± 3.5	24.4 ± 5.1
CD8	2.0 ± 1.1	2.3 ± 1.1	10.5 ± 1.0
CD25	4.9 ± 1.3 ^a^	1.0 ± 0.1	8.0 ± 1.1
CD152	14.2 ± 2.0 ^a^	4.6 ± 1.9	32.6 ± 4.3
CD19	3.3 ± 0.9	4.6 ± 0.5	3.5 ± 0.5
HLA-DR-DP	10.2 ± 1.5 ^a^	4.3 ± 1.8	12.1 ± 1.8
CD11b	1.1 ± 0.4	1.0 ± 0.3	1.4 ± 0.2
CD138	2.6 ± 0.4	1.4 ± 0.1	1.9 ± 0.1
CD80	2.2 ± 0.6	2.1 ± 0.9	1.4 ± 0.5
CD16	1.1 ± 0.2	1.0 ± 0.1	1.3 ± 0.1
CD56	1.4 ± 0.8	2.8 ± 0.3	4.3 ± 0.6

*Note*: The cells were stained using multicolor panels of the indicated surface markers and analyzed by flow cytometry. Data are expressed as mean ± SD (n = 10) of the percentage of specific immune-cell subsets relative to total leukocytes. Statistically significant differences vs. untreated cells (negative control) were determined using the Mann–Whitney U test (*p* < 0.001). ^a^ Values are statistically significant compared with untreated control (*p* < 0.001). The positive control (PHA-L) represents mitogen-induced T-cell activation.

**Table 2:** Antibacterial activity of *Phormidium ambiguum* extract against bacterial strains.

Bacterial Strains	*P. ambiguum* Extract (mm)	Antibiotics (mm)	DMSO/Water (1:1) (mm)
Gram-negative			
*Escherichia coli*	18.65 ± 1.24 ^a^	26.75 ± 1.45	0.00 ± 0.00
*Pseudomonas aeruginosa*	15.78 ± 0.96 ^a^	27.12 ± 1.20	0.00 ± 0.00
*Proteus vulgaris*	17.42 ± 1.10 ^a^	25.80 ± 1.34	0.00 ± 0.00
Gram-Positive			
*Bacillus subtilis*	14.68 ± 0.83 ^a^	24.50 ± 1.05	0.00 ± 0.00
*Micrococcus luteus* ATCC 49732	12.47 ± 0.91 ^a^	25.20 ± 0.98	0.00 ± 0.00
*Staphylococcus epidermidis* ATCC 49134	9.85 ± 0.72 ^a^	24.35 ± 1.16	0.00 ± 0.00

*Note*: Zones of inhibition (mm) are expressed as mean ± SD (n = 4). Penicillin/streptomycin and DMSO/water (1:1) were used as positive and negative controls, respectively. ^a^ Values statistically significant vs. control (*p* < 0.001).

**Table 3:** Putative bioactive compounds identified in the non-polar fraction of *P. ambiguum* extract by LC-ESI-MS/MS (positive ion mode).

No	RT [min]	Compound	Molecular Formula	Nature of Ion	Calculated *m*/*z*	Observed *m*/*z*	Error (ppm)	MS/MS Fragment Ions (*m*/*z*) *
1	10.55	Phomoarcherin B	C_23_H_28_O_5_	[M + H]^+^	385.1967	385.1973	+1.6	
2	10.66	Kampanol A	C_25_H_32_O_6_	[M + H]^+^	429.2272	429.2272	0.00	
3	11.2	Deoxyketomixol fucoside	C_46_H_64_O_7_	M•^+^	728.4652	728.4646	−0.83	581.3995 (−147.0657, loss of C_6_H_11_O_4_)
4	12.1	Mixol Methyl Fucoside	C_47_H_68_O_7_	M•^+^	744.4965	744.4982	2.28	525 (−58, loss of C_3_H_7_O and −161, loss of C_7_H_13_O_4_)
5	12.42	Nahuoic acid A	C_30_H_50_O_7_	[M + Na]^+^	545.3455	545.3462	+1.3	
6	13.59	Palmitic amide	C_16_H_33_NO	[M + H]^+^	256.2635	256.2635	0.00	
7	13.92	Oleic acid amide (Oleamide)	C_18_H_35_NO	[M + H]^+^	282.2791	282.2791	0.00	
8	14.94	Canthaxanthin	C_40_H_52_O_2_	[M + H]^+^	565.4051	565.4042	–1.6	
9	15.1	Zeaxanthin	C_40_H_56_O_2_	M•^+^	568.4280	568.4313	5.75	476 (−92, loss of toluene)
10	15.8	Oscillamid Y	C_45_H_59_N_7_O_10_	[M + H]^+^	858.4396	858.4396	0.00	
11	17.8	DGDG 16:2/18:2	C_49_H_84_O_15_	[M + H]^+^	913.5883	913.5883	0.00	733 (−180, loss of Hex; −197, loss of Hex + NH_3_); 571 (−162, loss of a dehydrated hexose residue [M + H−(Hex−H_2_O)] and −17, loss of NH_3_)
12	18.7	MGDG 16:2/18:2	C_43_H_74_O_10_	[M + H]^+^	751.5355	751.5355	0.00	571 (−162, loss of dehydrated galactose residue (Gal−H_2_O) and −17 loss of NH_3_); 589 (−180, loss of galactose moiety Gal, and −17 loss of NH_3_)
13	19.2	Phormidepistatin	C_52_H_84_N_12_O_16_	[M + H]^+^	1049.5004	1049.4992	–1.1	
14	20.5	Echinenone	C_40_H_54_O	[M + H]^+^	551.4247	551.4272	4.46	458.3 (−92, loss of toluene); 348.4 (−203, loss of C_14_H_19_O)
15	21.2	Chlorophyll a	C_55_H_72_O_5_N_4_Mg	M•^+^	892.5353	892.5370	1.89	617.2436 (−278 loss of phytyl side chain [M + H−C_20_H_38_]^+^); 555.2257 (−278, loss of phytyl side chain and −59, methoxycarbonyl group (–COOCH_3_))
16	24.65	Oligomycin C	C_45_H_74_O_10_	[M + Na]^+^	797.5177	797.5189	+1.5	
17	23.7	β-carotene	C_40_H_56_	[M + H]^+^	537.4455	537.4455	0.00	444.3780 (−92, loss of toluene); 456.3809 (−80, loss of methyl-cyclopentadiene); 429.3531 (−106, loss of xylene)
18	24.7	Oscillatoxin A	C_31_H_46_O_10_	[M + H]^+^	579.3164	579.3164	0.00	
19	25.52	Azithromycin	C_38_H_72_N_2_O_12_	[M + H]^+^	749.5158	749.5158	0.00	
20	29.07	Betulin	C_30_H_50_O_2_	[M + H]^+^	443.3884	443.3884	0.00	

* Fragmentation data are reported for selected representative metabolites, primarily pigments and polar lipid species, for which characteristic and literature-supported fragmentation pathways are well established and provide reliable structural confirmation (Figures S1–S10).

## Data Availability

The original contributions presented in this study are included in the article/Supplementary Material. Further inquiries can be directed to the corresponding authors.

## Supplementary Materials

The following supporting information can be downloaded at: https://www.mdpi.com/article/10.3390/plants15010033/s1, Figure S1: LC–ESI–MS extracted ion chromatograms of identified pigments in *Phormidium ambiguum*; Figure S2: MS/MS spectrum of the ion at *m*/*z* 728.4666; Figure S3: MS/MS spectrum of the ion at *m*/*z* 744.4984; Figure S4: MS/MS spectrum of the ion at *m*/*z* 568.4302; Figure S5: MS/MS spectrum of the ion at *m*/*z* 551.4270; Figure S6: MS/MS spectrum of the ion at *m*/*z* 892.5375; Figure S7: MS/MS spectrum of the ion at *m*/*z* 536.4402; Figure S8: LC–ESI–MS extracted ion chromatograms of identified polar lipids in *P. ambiguum*; Figure S9: MS/MS spectrum of the ion at *m*/*z* 930.6163; Figure S10: MS/MS spectrum of the ion at *m*/*z* 768.5635.
