# Supplementary material for: Cyanobacterial Biofactories Beyond Model Strains: Exploratory Screening of Immunomodulatory Activity in Phormidium ambiguum Extracts
Source: Plants (Basel). 2025 Dec 22;15(1):33. doi: 10.3390/plants15010033 (PMC12787963; doi:10.3390/plants15010033)
Supplement: Supplementary file 1 [file plants-15-00033-s001.zip › plants-4017372-supplementary.pdf]

Supplementary S1. MS/MS fragmentation data of selected metabolites tentatively identified in the non-polar fraction of *Phormidium ambiguum* extract by UHPLC–ESI–QTOF–MS/MS (positive ion mode).

A. Pigments

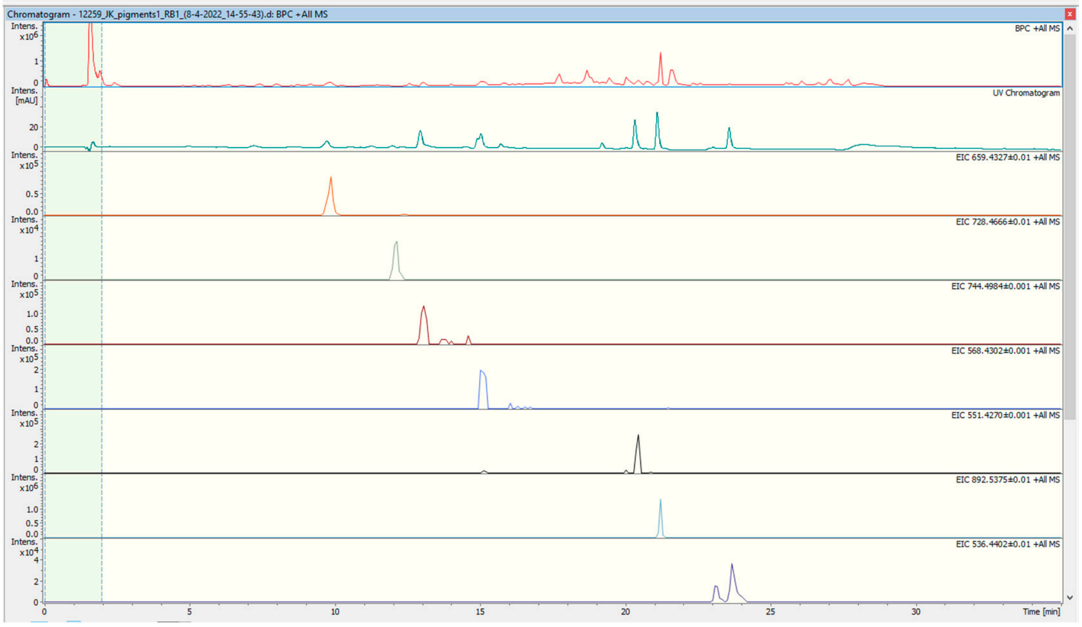

Figure S1. LC–ESI–MS extracted ion chromatograms of identified pigments in *Phormidium ambiguum*

Peak 1. retention time 11.2 min.

Deoxyketomyxol Fucoside mass=728.4652

Deoxymyxol Metyl Fucoside mass=728.501590

m/z 728.4666; formula= $C_{46}H_{64}O_{7;}$

Structure and proposed fragmentation of the Deoxyketomyxol Fucoside.

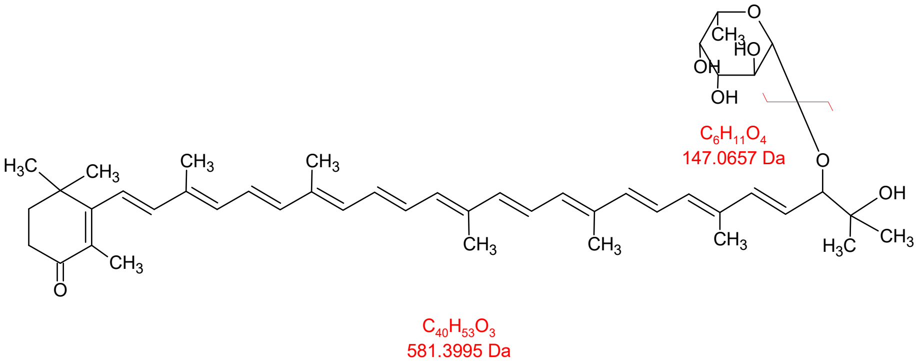

MS/MS 728.4666

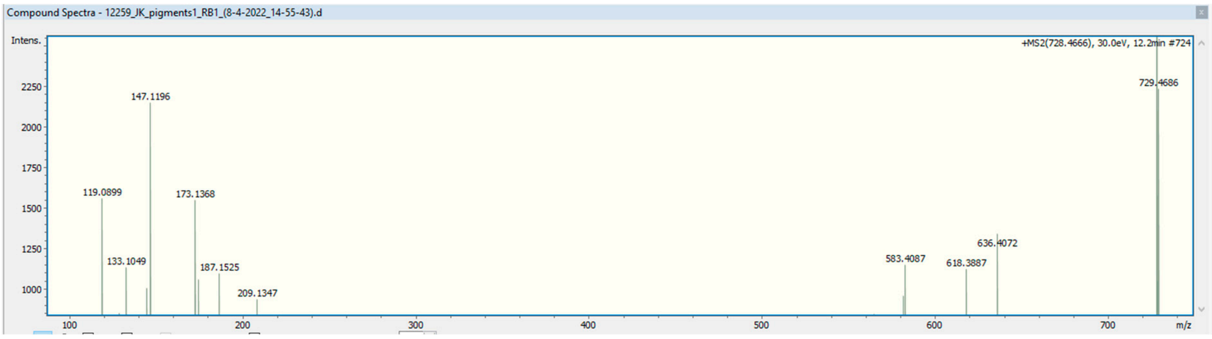

Figure S2. MS/MS spectrum of the ion at m/z 728.4666

- The precursor ion was detected at m/z 728.4666, consistent with the protonated molecule $[M+H]^+$ of deoxyketomixol fucoside ($C_{46}H_{64}O_7$), with a mass accuracy within acceptable limits for high-resolution Q-TOF analysis.
- MS/MS fragmentation of the precursor ion at m/z 728.4666 produced several diagnostic fragment ions. A prominent fragment ion observed at m/z 581.3995 corresponds to the aglycone-related ion formed after cleavage of the glycosidic bond, consistent with the loss of a hexose moiety. Additional low-mass fragment ions at m/z 147.1196, 173.1368, and 187.1525 are attributed to characteristic sugar-derived fragments, supporting the presence of a fucosyl residue. Higher-mass fragment ions detected at m/z 618.3887 and 636.4072 likely arise from partial rearrangements or secondary cleavages within the carotenoid backbone.

Peak 2. retention time 12.1 min. (Myxol Metyl Fucoside)

m/z 744.4984; **Myxol Metyl Fucoside:** formula= $C_{47}H_{68}O_7$; mass= 744.496505

Structure and proposed fragmentation of the Myxol Metyl Fucoside.

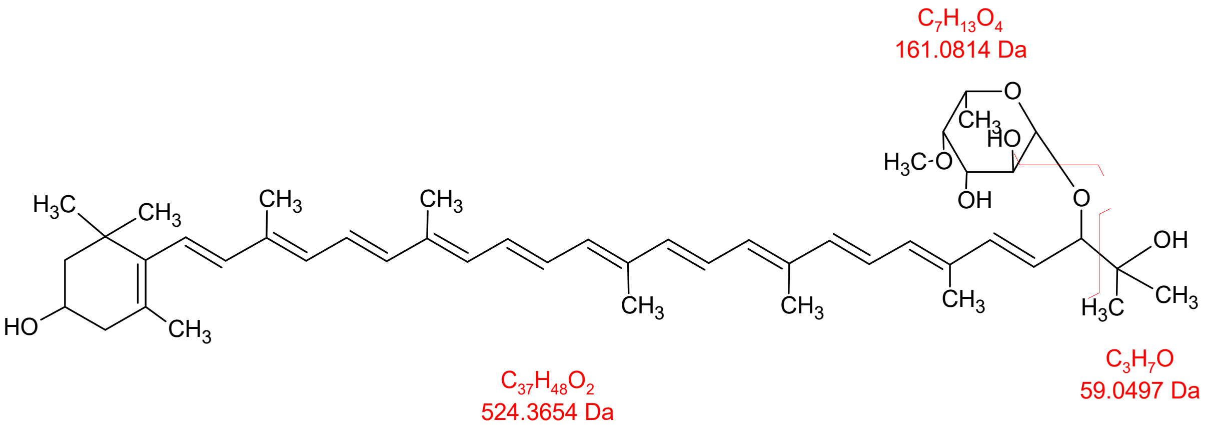

MS/MS 744.4984

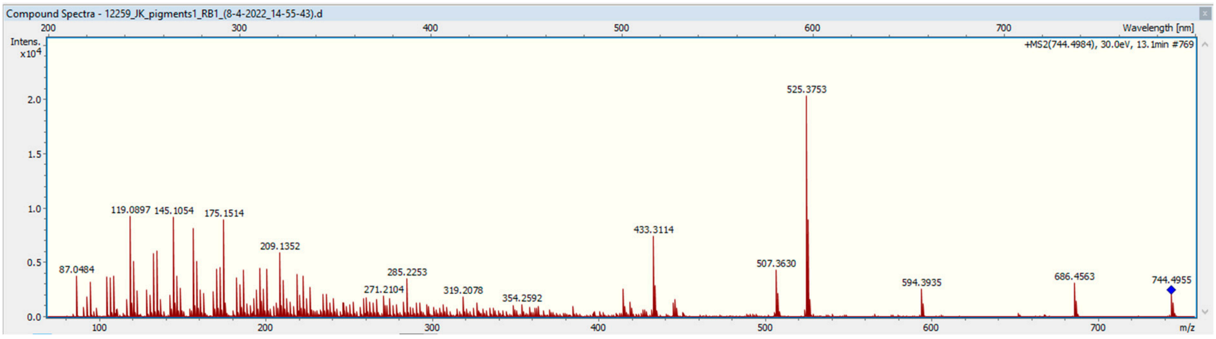

Figure S3. MS/MS spectrum of the ion at m/z 744.4984

- The precursor ion was detected at m/z 744.4984, corresponding to the protonated molecule $[M+H]^+$ of myxol methyl fucoside ($C_{47}H_{68}O_7$), with mass accuracy consistent with high-resolution Q-TOF measurements.
- MS/MS fragmentation of the precursor ion at m/z 744.4984 yielded several characteristic fragment ions. A prominent fragment ion observed at m/z 686.4563 is assigned to the loss of a methoxy group from the fucosyl moiety, a fragmentation commonly reported for methylated glycosylated carotenoids. The most intense fragment ion at m/z 525.3753 corresponds to the aglycone-related ion formed by cleavage of the glycosidic bond and subsequent rearrangement within the polyene backbone. An additional fragment ion detected at m/z 507.3630 is attributed to further elimination of a small neutral molecule from the aglycone fragment.

- Low-mass fragment ions in the range m/z 119–175 are consistent with sugar-derived fragments, supporting the presence of a fucosyl substituent.

Peak 3. retention time 15.1 min. (Zeaxanthin)

m/z 568.4302; **Zeaxanthin:** formula= $C_{40}H_{56}O_2$; mass= 568.42803;

Structure and proposed fragmentation of the Zeaxanthin.

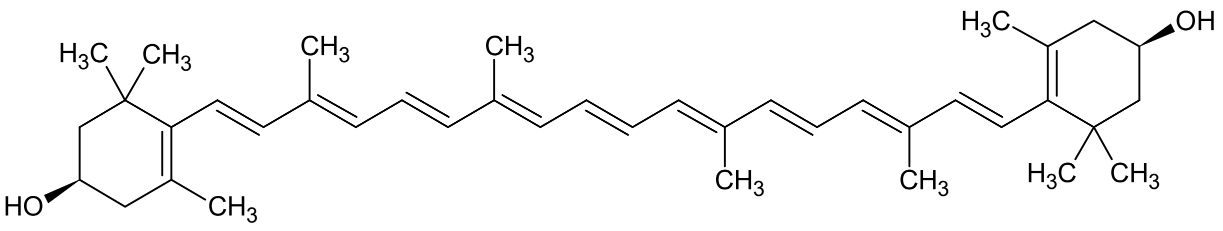

MS/MS 568.4302

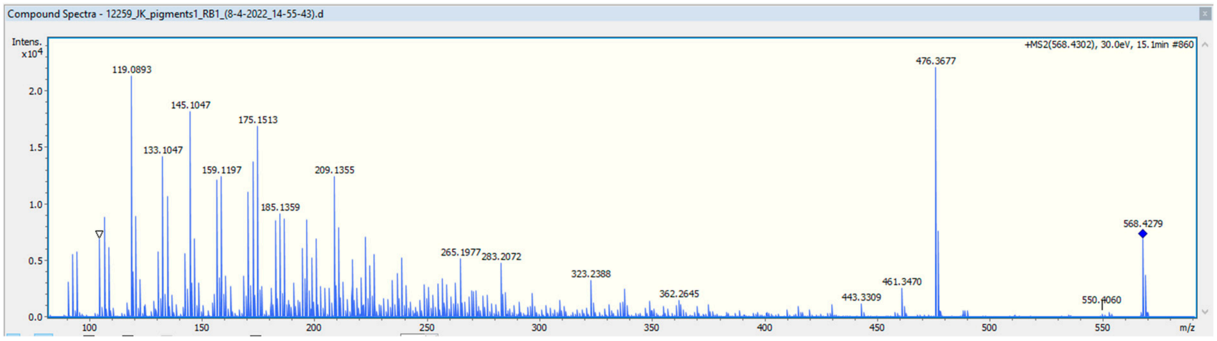

Figure S4. MS/MS spectrum of the ion at m/z 568.4302

- The precursor ion was detected at m/z 568.4302, corresponding to the protonated molecule $[M+H]^+$ of zeaxanthin ($C_{40}H_{56}O_2$), with mass accuracy consistent with high-resolution Q-TOF analysis.
- MS/MS fragmentation of the precursor ion at m/z 568.4302 produced several characteristic fragment ions. A fragment ion observed at m/z 550.4050 is attributed to dehydration of the parent molecule, a common fragmentation pathway for hydroxylated carotenoids. The most intense fragment ion detected at m/z 476.3677 corresponds to cleavage within the polyene chain, consistent with the elimination of a C_7H_8 unit from the conjugated backbone. This fragmentation behavior is well documented for xanthophylls and reflects the instability of extended polyene systems under collision-induced dissociation.

Peak 4. retention time 20.5 min. (Echinenon)

m/z 551.4270; **Echinenon:** formula= $C_{40}H_{54}O$; mass= 550.4;

Fragmentation 551 ⟶ 458.3 (-92, loss of toluen), (-203, loss of $C_{14}H_{19}O$)

Structure and proposed fragmentation of the Echinenon.

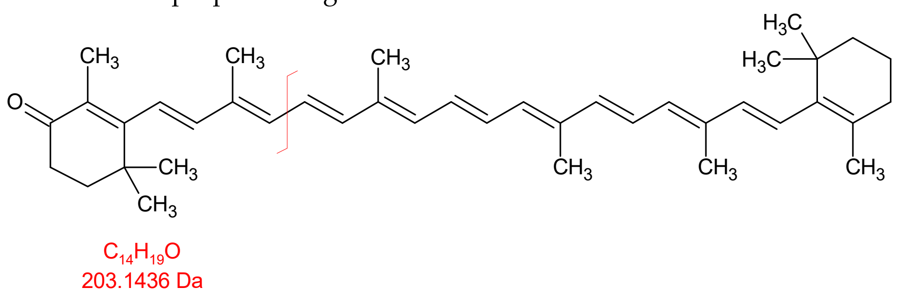

MS/MS 551.4270

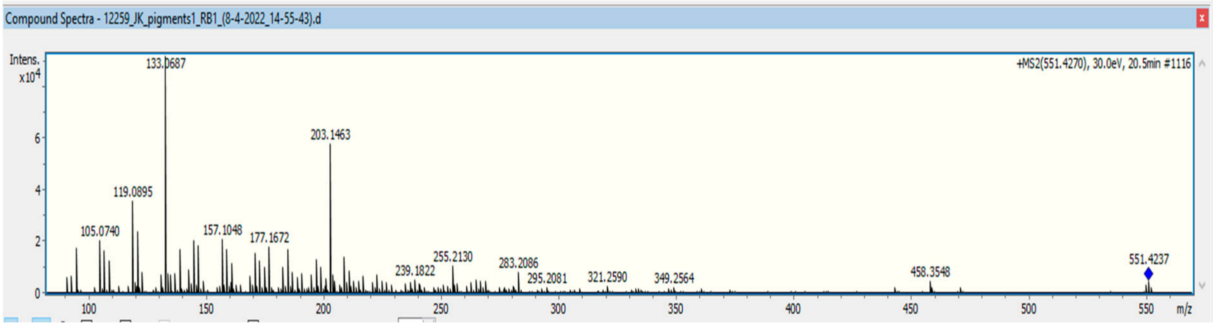

Figure S5. MS/MS spectrum of the ion at m/z 551.4270

- The precursor ion was detected at m/z 551.4270, corresponding to the protonated molecule $[M+H]^+$ of echinenone ($C_{40}H_{54}O$), in agreement with its theoretical exact mass.
- MS/MS fragmentation of the precursor ion at m/z 551.4270 yielded several characteristic fragment ions. A prominent fragment ion observed at m/z 458.3548 is attributed to cleavage within the conjugated polyene chain, consistent with the elimination of a C_7H_8 unit from the carotenoid backbone. This type of fragmentation is typical for keto-carotenoids under collision-induced dissociation.
- Another abundant fragment ion detected at m/z 348.2954 corresponds to a deeper fragmentation of the polyene system involving loss of a larger aliphatic moiety from the terminal ring region. Additional lower-mass fragment ions in the range m/z 105–205 arise from successive cleavages and rearrangements within the conjugated system and cyclic end groups.

Peak 5. retention time 21.2 min. (Chlorophyll a.)

m/z 892.5375 assigned to protonated **Chl. a** $[M+H]^+$, molecular formula= $C_{55}H_{72}O_5N_4Mg$;

Structure and proposed fragmentation of the Chl a.

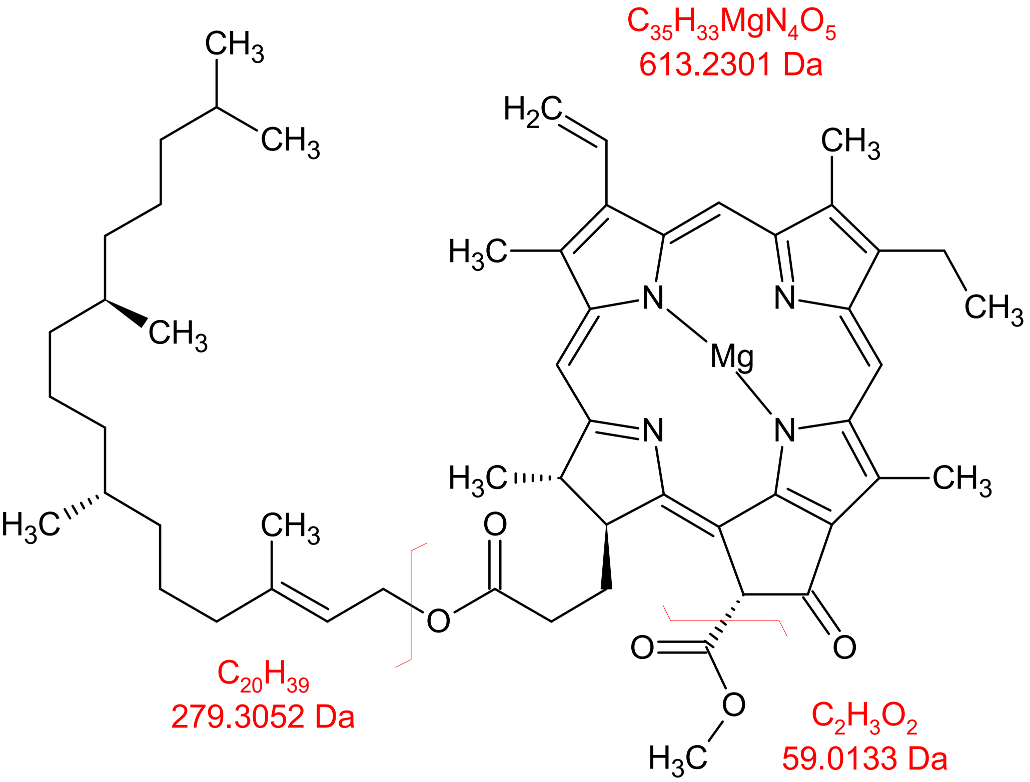

MS/MS 892.5375

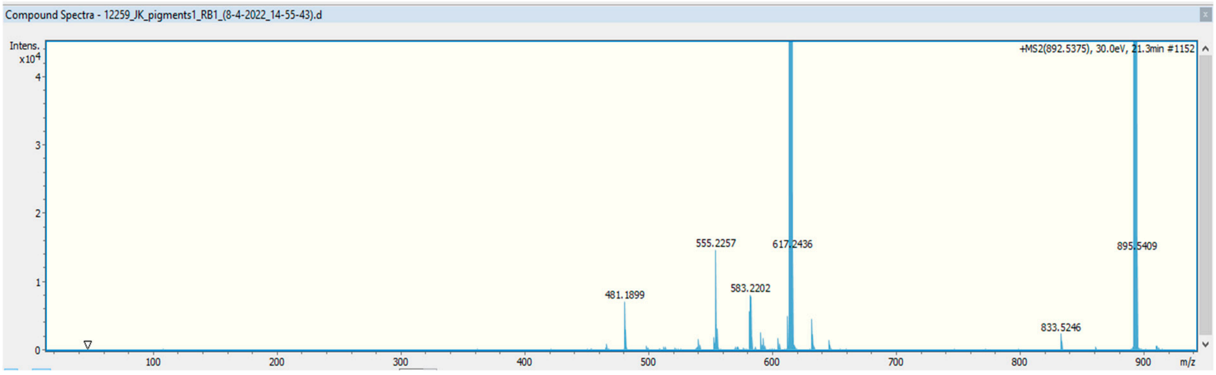

Figure S6. MS/MS spectrum of the ion at m/z 892.5375

- The MS/MS spectrum of the precursor ion at m/z 892.5375 exhibited characteristic product ions consistent with previously reported fragmentation pathways of chlorophyll a under positive ESI conditions.
- A prominent fragment ion at m/z 617.2436 was observed and is assigned to the loss of the phytyl side chain, corresponding to a neutral loss of 278 Da, yielding the porphyrin core fragment $[M+H–C_{20}H_{38}]^+$. This fragmentation is well documented as a diagnostic feature of chlorophyll a and related chlorophyll derivatives.
- An additional fragment ion at m/z 555.2257 can be rationalized by the combined neutral loss of the phytyl chain (278 Da) and a methoxycarbonyl group ($–COOCH_3$, 59 Da), forming the ion $[M+H–C_{20}H_{38}–COOCH_3]^+$. The loss of the methoxycarbonyl substituent from the chlorin macrocycle is a commonly reported secondary fragmentation pathway.
- A lower-mass fragment ion at m/z 481.1899 is attributed to further fragmentation of the chlorin ring system, involving sequential neutral losses from the macrocycle following phytyl and methoxycarbonyl cleavage. Such product ions are frequently observed in high-energy MS/MS spectra of chlorophyll a and reflect progressive degradation of the tetrapyrrole structure.

Peak 6. retention time 23.7 min.

m/z 536.4402; **β-Carotene**: formula= $C_{40}H_{56}$; mass= 536.4;

The most informative ions in MS/MS spectrum of the β-carotene are at *m/z* 444,3780 (loss of toluene -92 Da), *m/z* 456.3809 (loss of methyl-cyclopentadiene -80 Da), 429.3531 (loss of xylene -106 Da) and *m/z* 412.3210 (loss of one of the rings -123 Da).

Structure and proposed fragmentation of the β-Carotene.

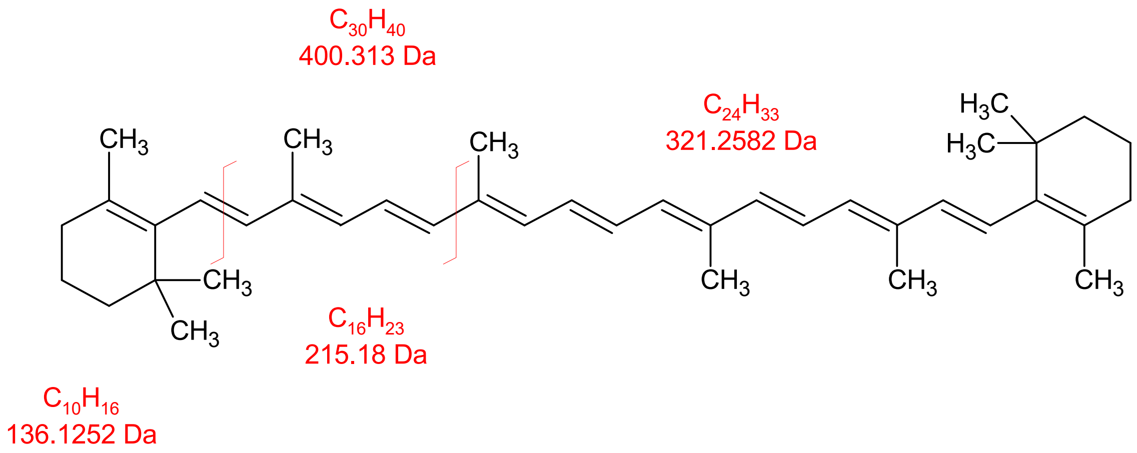

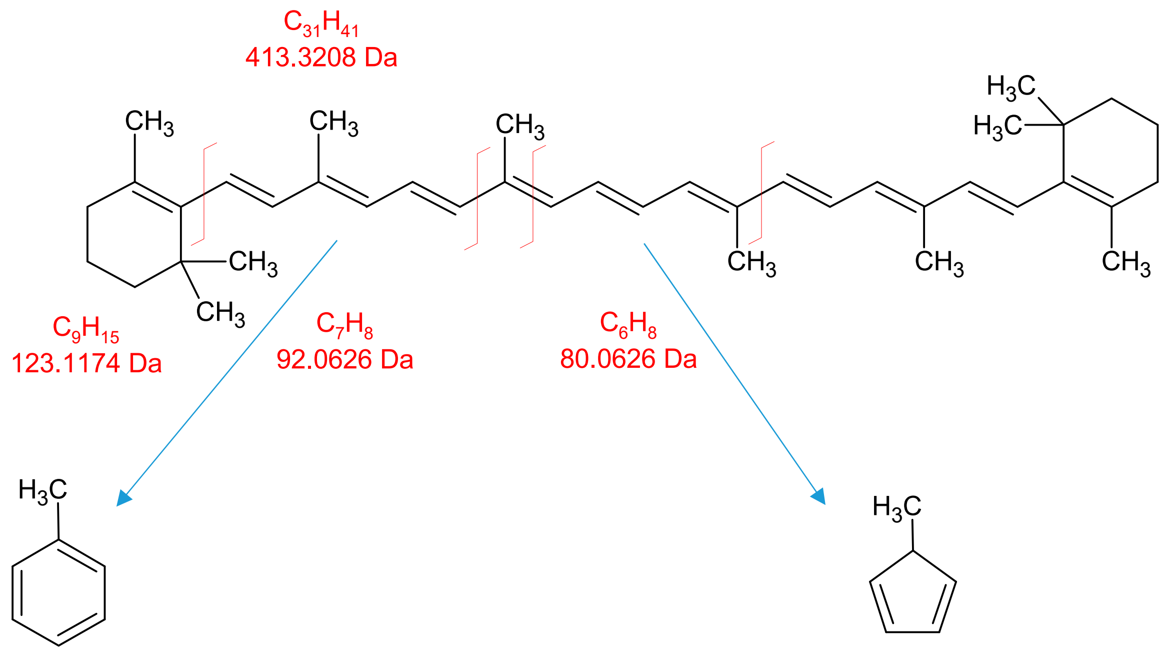

Toluene, 92.0626 Da

Methyl-cyclopentadiene, 80.0626 Da

MS/MS 536.4402

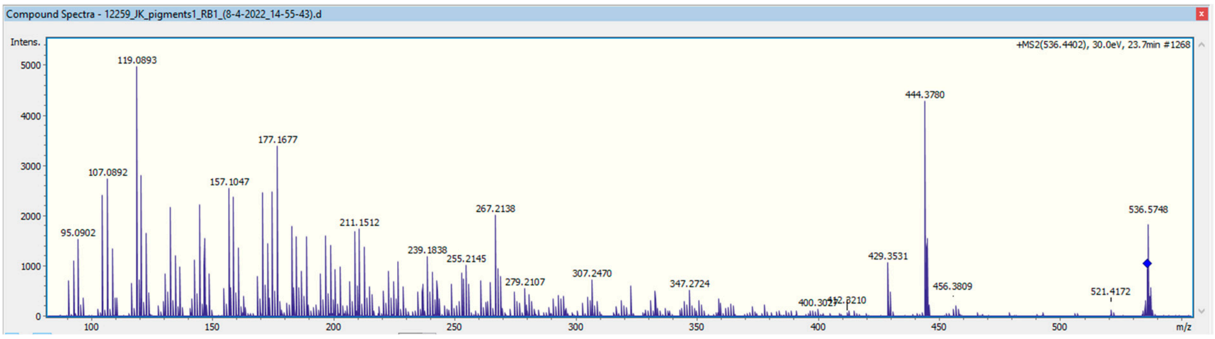

Figure S7. MS/MS spectrum of the ion at m/z 536.4402

The MS/MS spectrum of β-carotene ([M]•⁺, *m/z* 536.4402) displayed characteristic fragment ions at *m/z* 444.3780, 456.3809, 429.3531, and 412.3210. These ions arise from stepwise cleavage of the polyene chain and loss of aromatic and cyclic substructures, consistent with previously reported fragmentation of carotenoid radical cations.

B. Polar Lipids

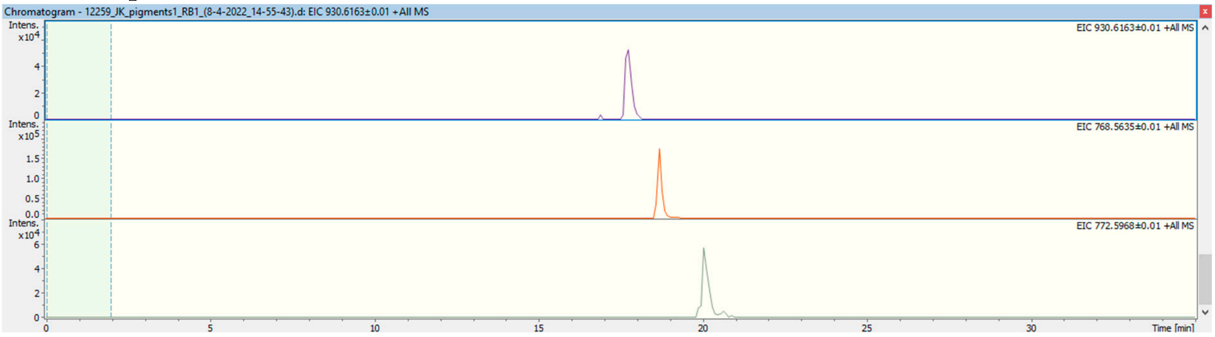

Figure S8. LC–ESI–MS extracted ion chromatograms of identified polar lipids in *P. ambiguum*

Peak 1. retention time 17.8 min.

m/z 930.6163 $[M+NH_4]^+$; **DGDG 16:2/18:2:** formula= $C_{49}H_{84}O_{15}$; mass= 912,581;

MS/MS 930.6163

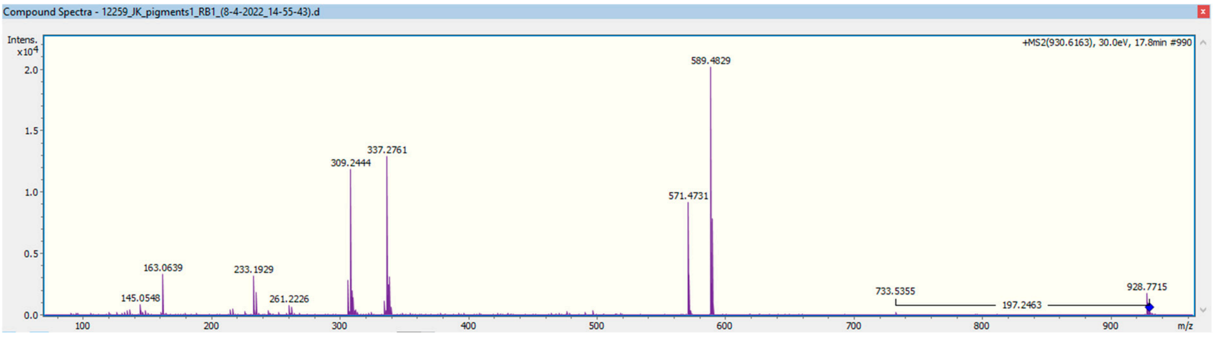

Figure S9. MS/MS spectrum of the ion at m/z 930.6163

Fragmentation of the galactolipid precursor ion was observed predominantly from the ammonium adduct $[M+NH_4]^+$, which is typical for neutral glycolipids analyzed in positive ESI mode.

- The fragment ion at m/z 733 corresponds to a neutral loss of 197 Da from the $[M+NH_4]^+$ precursor. This loss is consistent with the simultaneous elimination of a hexose moiety (180 Da) and ammonia (17 Da), yielding the ion $[M+H-Hex]^+$. Given the lipid class under investigation (DGDG), this fragmentation is assigned to the cleavage of a galactosyl residue, in agreement with the known galactolipid structure of cyanobacterial membranes.
- The ion at m/z 589 arises from the combined loss of two hexose units together with ammonia from the ammonium adduct precursor, corresponding to $[M+NH_4-2Hex-NH_3]^+$, which is equivalent to $[M+H-2Hex]^+$. This fragmentation pathway is characteristic of digalactosyldiacylglycerols and reflects sequential cleavage of glycosidic bonds.
- The fragment detected at m/z 571 represents a neutral loss of 179 Da, corresponding to the elimination of a dehydrated hexose residue (162 Da) together with ammonia (17 Da). This ion is therefore assigned to $[M+H-(Hex-H_2O)]^+$, a well-documented fragmentation route for sugar-containing lipids under positive ESI conditions.
- Acyl-chain–related fragment ions were also observed and provided diagnostic information on the fatty acid composition of the galactolipid backbone. The fragment at m/z 309, corresponding to R_1CO + 74, is indicative of a hexadecadienoic acid (C16:2) moiety, while the ion at m/z 337 (R_2CO + 74) is characteristic of a linoleic acid (C18:2) residue. These fragments are consistent with charge-remote fragmentation of the glycerol backbone and align with fatty acid profiles commonly reported for cyanobacterial galactolipids.

Peak 2. retention time 18.7 min.

m/z 768.5635 $[M+NH_4]^+$; **MGDG 16:2/18:2:** formula= $C_{43}H_{74}O_{10}$; mass= 750.5282

MS/MS 768.5635

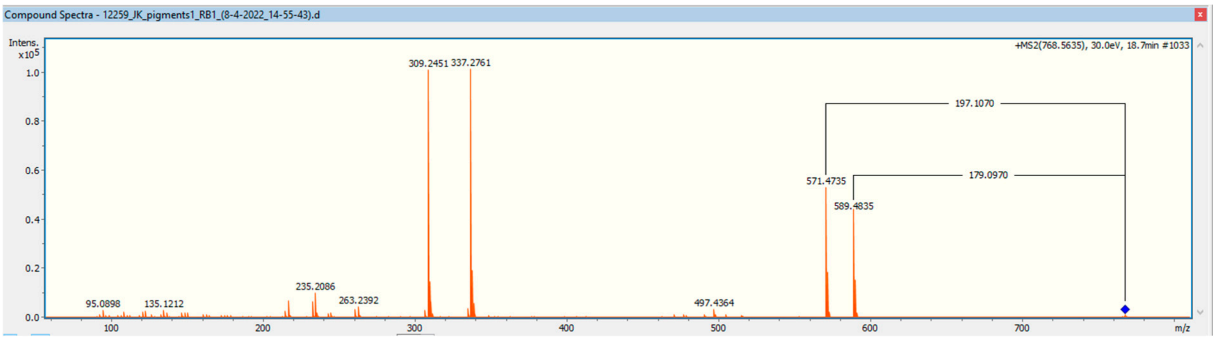

Figure S10. MS/MS spectrum of the ion at m/z 768.5635

Fragmentation of the ammoniated precursor ion *$[M+NH_4]^+$* generated characteristic product ions consistent with the galactolipid structure of MGDG.

- The fragment at m/z 589 corresponds to a neutral loss of 197 Da, which is assigned to the combined elimination of a galactose moiety (Gal, 180 Da) and ammonia (NH_3, 17 Da), yielding the ion *$[M+H{-}Gal]^+$*. This fragmentation pathway is well documented for ammonium-adducted galactolipids and supports the presence of a hexose headgroup.
- The ion observed at m/z 571 reflects a neutral loss of 179 Da, attributed to the simultaneous loss of a dehydrated galactose residue *(Gal–H_2O,162 Da)* and ammonia *(NH_3, 17 Da)*, resulting in the fragment *$[M+H{-}(Gal{-}H_2O)]^+$*. Such a loss is characteristic of glycosylated lipids and represents a common dehydration pathway of hexose moieties under collision-induced dissociation.
- In addition, acyl-related diagnostic fragment ions were detected at m/z 309 and m/z 337, corresponding to $R_1CO + 74$ and $R_2CO + 74$, respectively. These fragments are indicative of the fatty acyl substituents and are assigned to a hexadecadienoic acid (C16:2) and a linoleic acid (C18:2) residue. The presence of these ions confirms the acyl chain composition of the MGDG molecule and is consistent with previously reported MS/MS behavior of cyanobacterial galactolipids.
